# Selective Copper(II) Complexes against *Mycobacterium tuberculosis*

**DOI:** 10.1021/acsomega.5c10934

**Published:** 2025-12-29

**Authors:** Kaíque A. D’Oliveira, Nícolas Glanzmann, Adilson D. da Silva, Carlos E. T. Bruzeguini, Marcos A. Ribeiro, Christian S. Carnero Canales, Cesar A. Roque-Borda, Fernando R. Pavan, Débora F. M. da Silva, Douglas H. Pereira, Alexandre Cuin

**Affiliations:** † Laboratório de Química Bioinorgânica, Department of Chemistry, Institute of Exact Sciences, 28113 Federal University of Juiz de Fora (UFJF), Juiz de Fora, Minas Gerais 36036-330, Brazil; ‡ Department of Chemistry, Institute of Exact Sciences, Federal University of Juiz de Fora (UFJF), Juiz de Fora, Minas Gerais 36036-330, Brazil; § Department of Chemistry, Institute of Exact Sciences, 28126 Federal University of Espírito Santo (UFES), Vitória, ES 29075-910, Brazil; ∥ School of Pharmaceutical Sciences, Department of Biological Sciences, São Paulo State University (UNESP), Araraquara, SP 14800-900, Brazil; ⊥ School of Pharmacy, Biochemistry and Biotechnology, Santa Maria Catholic University, Arequipa 04013, Perú; # Technological Institute of Aeronautics (ITA), São José dos Campos, São Paulo 12228-900, Brazil

## Abstract

The present paper describes the synthesis, characterization, and biological activity of five quinoline derivatives, DCQ; ACQ12; ACQ13; ACQ14; ACQophen, and their respective copper­(II) complexes. The class of organic compounds is composed of 4,7-dichloroquinoline (DCQ) and its derivatives containing aliphatic diamines, 1,2-ethanediamine (ACQ12); 1,3-propanediamine (ACQ13); 1,4-butanediamine (ACQ14); and an aromatic diamine, *o*-phenylenediamine (ACQophen), as a side chain at the 4-position of the quinoline ring. Single-crystal X-ray diffraction was used to determine the structure of the Cu-DCQ complex (1:2 M:L ratio), while the structure of the Cu-ACQ12 complex (1:1 M:L ratio) was obtained from powder X-ray diffraction (PXRD) data. Spectroscopic (IR, Raman, UV–vis) and analytical data supported coordination through the quinoline nitrogen atom in all complexes. DFT (M06-2X/6-31G) calculations complemented the experimental results, revealing distinct coordination geometries and molar ratios: Cu-ACQ13 and Cu-ACQ14 exhibit a 1:1 (M:L) stoichiometry with distorted square-planar Cu­(II) centers, while Cu-ACQophen crystallizes in a 1:2 (M:L) ratio featuring a slightly elongated square-pyramidal geometry, consistent with nonelectrolytic behavior in DMSO. Theoretical vibrational frequencies showed good agreement with experimental spectra, validating the proposed models. The organic and inorganic compounds described here showed potent activity against *Mycobacterium tuberculosis* (*Mtb*) and selectivity indexes. The Cu-ACQophen complex exhibited relevant antitubercular activity with a low MIC_90_ value, about 1.68 μmol L^**–**1^, and a high selectivity index, SI = 48. Complexes Cu-DQC and Cu-ACQ12 also demonstrated SI values >10.

## Graphical abstract

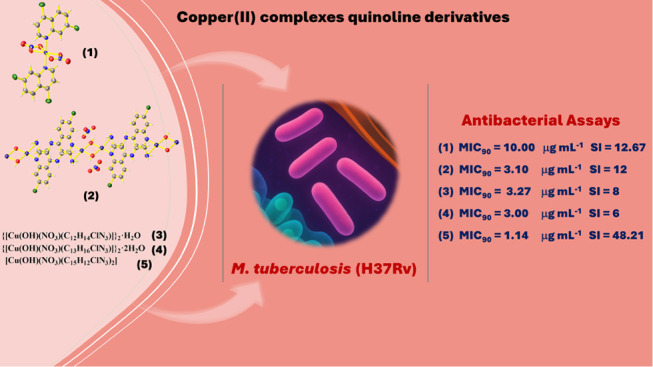

## 1 Introduction

Tuberculosis, TB, is a serious infectious disease caused by the bacteria *Mycobacterium tuberculosis* (*Mtb)* which mainly affects the lungs but can also affect other organs in our body.^1^ Its transmission occurs from person to person through droplets of saliva expelled when people talk, cough, or sneeze.^2^ Despite being an ancient disease, dating back 9000 years ago for first documented appearance in human society,^3,4^ Tuberculosis remains a major public health challenge, especially in countries with more vulnerable health systems.^5^ Tuberculosis treatment requires the combined administration of antimicrobial agents over a prolonged period, about 6 months for cases of drug-sensitive pulmonary tuberculosis and 9 to 20 months for drug-resistant tuberculosis.^6^ TB control depends on coordinated efforts, including early diagnosis, access to effective medicines, and prevention strategies, with awareness and adequate treatment being essential to fight TB, which is still prevalent in many regions of the world.^7,8^

One promising class of compounds under investigation against *Mtb* strains comprises quinoline derivatives, particularly 7-chloro-4-aminoquinolines synthesized from the 4,7-dichloroquinoline scaffold (Figure 1), which exhibit notable antimicrobial, antiparasitic,^9–11^ and mycobactericidal activities.^12–15^ In some cases, these compounds have shown efficacy against *M. tuberculosis*, although their evaluation remains at the *in vitro* preclinical stage.^16,17^ Their antimicrobial activity is attributed, in part, to their accumulation in acidic organelles such as the digestive vacuoles of parasites and lysosomes of infected host cells, where they interfere with vital cellular processes. The core structure of 7-chloro-4-aminoquinoline offers multiple sites amenable to chemical modification, enabling the development of analogues with enhanced biological activity, improved selectivity, and reduced cytotoxicity.^18–20^ The structure–activity relationship is closely associated with the chlorine atom at position 7, as well as the polarity of the side chain at position 4 relative to the quinoline ring.^16,21^

**Figure 1:**
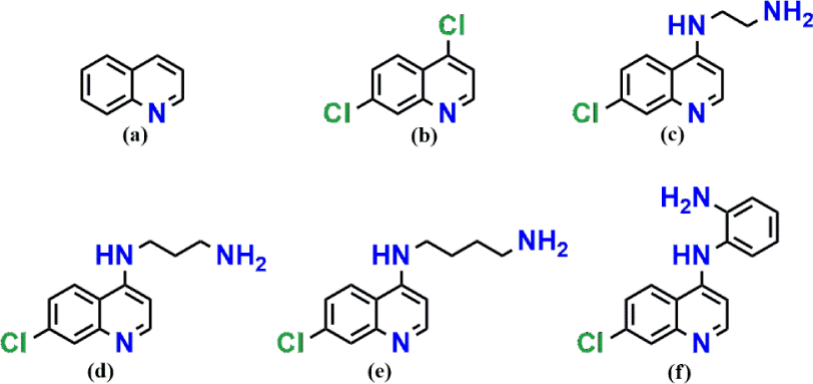
Schematic structures of the quinoline nucleus (a), 4,7-dichloroquinoline—DCQ (b), [*N* ^1^-(7-chloroquinolin-4-yl)­ethane-1,2-diamine]—ACQ12 (c), [*N* ^1^-(7-chloroquinolin-4-yl)­propane-1,3-diamine]—ACQ13 (d), [*N* ^1^-(7-chloroquinolin-4-yl)­butane-1,4-diamine]—ACQ14 (e), and [*N* ^1^-(7-chloroquinolin-4-yl)­benzene-1,2-diamine]—ACQophen (f).

Another strategy to enhance the biological activity of bactericidal agents involves incorporation of metal ions into their structures. The therapeutic potential of inorganic compounds has been extensively investigated and validated through scientific studies in the last century, when researchers began reporting the growing use of metal-based agents to combat bacteria, parasites, tumors, and fungi.^22,23^ Notable examples include the discovery of cisplatin, [Pt­(NH_3_)_2_Cl_2_], a widely used chemotherapeutic agent for various types of cancer; silver sulfadiazine, [Ag­(C_10_H_9_N_4_O_2_S)]_n_, applied in the prevention and treatment of bacterial infections in dermatological wounds; copper­(II) coordination compounds from the Casiopeina family with antineoplastic properties; and ruthenium­(II) complexes exhibiting promising anticancer activity.^24–27^ Within this context, copper­(II) complexes incorporating quinoline-based ligands have emerged as promising candidates against a broad range of pathogens, including *Mtb*, while also displaying antineoplastic potential. Their diverse biological activities are commonly credited to the accessible Cu­(II)/Cu­(I) redox cycling, structural versatility, and the ability to engage biological targets through both covalent coordination modes and noncovalent interactions.^28–32^

Previous studies by our group have demonstrated that halogenated quinolones, particularly 4,7-dichloroquinoline (DCQ) and 7-chloro-4-aminoquinoline derivatives (ACQ), readily form stable mononuclear and dimeric complexes with Silver­(I) ions,^33,34^ displaying relevant antitubercular properties. Within the broader field of medicinal inorganic chemistry, this study aligns with ongoing efforts to use coordination chemistry as a basis for developing new biologically active metal-containing compounds.^35–38^

Copper­(II) complexes supported by quinoline ligands were selected due to their consolidated antimicrobial and pharmacological profiles. Accordingly, the present work reports the synthesis, structural characterization, and biological evaluation of a new series of Cu­(II)-quinoline complexes (Figure 2) with the aim to elucidate their coordination features and assess their potential as novel candidates against *Mycobacterium tuberculosis*.

**Figure 2:**
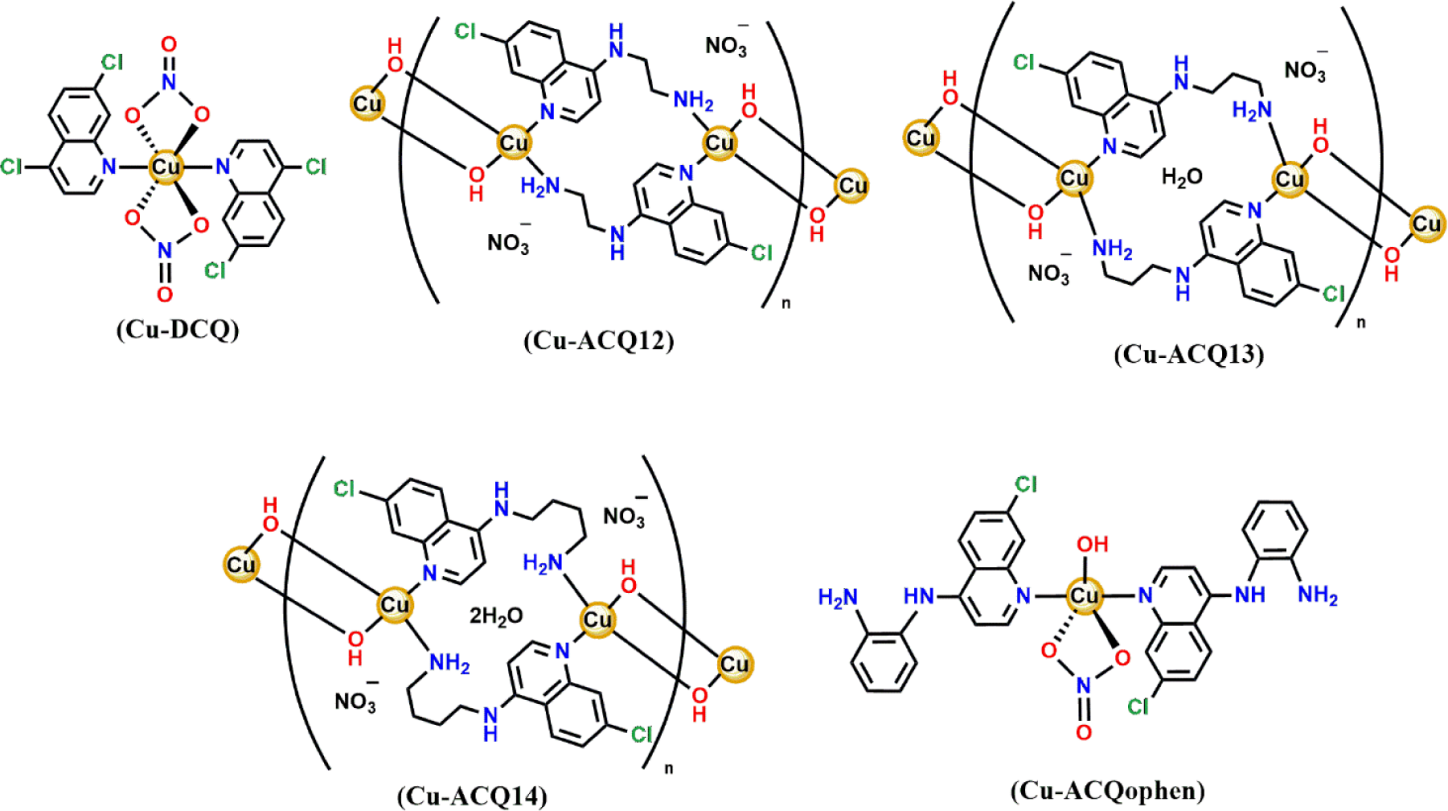
Structures of Cu-DCQ and Cu-ACQ12, by X-ray studies and DFT-proposed structures of Cu-ACQ13, Cu-ACQ14, and Cu-ACQophen complexes.

## 2 Experimental Section

### 2.1 General Considerations

The synthesis of the 7-chloro-4-aminoquinoline ligands was conducted according to the methodology previously described by D’Oliveira et al. (2025).^33^ The synthesis and purity of 7-chloro-4-aminoquinoline ligands also were confirmed by ^1^H NMR spectroscopy, whose spectra displayed the expected characteristic signals for each structure (SI—Figures S31 and S32). All chemicals and solvents were purchased and used without further purification. The solvents used in the syntheses of the ligands were ethanol (99.8%, Synth), ethyl ether (95.0%, Synth), hexane (95.0%, Synth), dichloromethane (100%, Synth), and chloroform (99.8%, Synth). The reagents used in the organic syntheses were: 4,7-dichloroquinoline (99%, CAS: 86-98-6), 1,2-diaminoethane (≥99%, CAS: 107-15-3), 1,3-diaminopropane (≥99%, CAS: 109-76-2), 1,4-diaminobutane (99%, CAS: 110-60-1), 1,2-diaminobenzene (99.5%, CAS: 95-54-5), sodium hydroxide pellets (≥97%, CAS: 1310-73-2, Synth), sodium sulfate (≥99%, CAS: 7757-82-6, Synth), silver nitrate (99%, CAS: 7761-88-8, Sigma-Aldrich), and Amberlite IRA-400 chloride form (CAS: 60177-39-1, Sigma-Aldrich).

For the synthesis of the Cu­(II) complexes, the solvents/reagents used were methanol (CH_3_OH) (99.8%, Synth), ethyl ether (Et_2_O) (95.0%, Synth), and acetonitrile (CH_3_CN) (99.5%, Êxodo Científica). Copper­(II) nitrate trihydrate (99%, CAS: 10031-43-3) was purchased from Sigma-Aldrich.

Elemental analyses (CHN) were performed on a PerkinElmer Series II 2400 CHNS/O system. Attenuated Total Reflectance Fourier-Transform Infrared spectra, ATR-FTIR, were recorded on a Bruker FT-IR Platinum ATR spectrophotometer in the 4000–400 cm^–1^ range, with a resolution of 4 cm^–1^ and 1024 scans, at 293 ± 1 K, for all compounds. Fourier-Transform Raman spectra, FT-Raman, were recorded on a Bruker RFS 100 FT-Raman spectrophotometer, λ_0_ = 1064 nm (Nd:YAG), in the 4000–50 cm^–1^ range, with a resolution of 4 cm^–1^, using 10 mW laser power, 1024 scans, and at 293 ± 1 K, for all compounds. UV–vis experiments were carried out on an Ocean Optics fiber USB 2000 spectrophotometer, DH-2000-BAL, operating with a Deuterium-Halogen laser at 254 and 700 nm wavelength, within the 200–1100 nm range, at 293 ± 1 K, using a quartz cuvette (1 cm, 2 mL), integration time 1 μs, to measure the maximum absorption wavelength with an absorbance below 1, for all compounds. ^1^H NMR spectra were measured on a Bruker Avance III HD 500 MHz spectrometer in deuterated dimethyl sulfoxide (DMSO-*d*_6_), at 297 K. The NMR spectra were obtained using 20 mg for all 7-chloro-4-aminoquinolines. The chemical shifts, δ, were reported in parts per million (ppm) and are referenced to the tetramethylsilane peak (TMS). Coupling constants (*J*) were measured in hertz (Hz). Melting points were determined with an MQAPF/Microquímica apparatus. Molar conductivities (Λ_M_) were obtained using MS Tecnopon-mCA 150, platinum sensor (*K* = 1 cm^–1^), Conductivity Standard KCl (146.9 μS cm^–1^ ± 0.5% at 298 ± 0.2 K). The molar conductivities of complexes were obtained in 25 mL DMSO solutions, 1.0 × 10^–3^ mol L^–1^, and evaluated at specific intervals during a 24 h period at room temperature (RT), 298 ± 2 K.

### 2.2 Single Crystal Method of Complex Cu-DCQ

Single crystal X-ray diffraction measurements were performed on an Agilent SuperNova diffractometer. Measurements were performed using the source of radiation CuKα, λ = 1.54184 Å. Data integration and scaling of reflection intensities were performed in the CrysAlis PRO 1.171.41.93a (Rigaku OD, 2020). The crystal structure of complex Cu-DQQ was solved using program SHELXT 2018/2,^39^ and SHELXL 2018/3 ^40^ was used for refinement applying the least-squares method. The structure of Cu-DCQ was drawn using the OLEX2 program.^41^

### 2.3 Powder Diffraction of Complexes Cu-ACQs

Full X-ray diffraction data (Supporting Information, Figures S49–S52) of polycrystalline compounds Cu-ACQs, meaning Cu-ACQ12, Cu-ACQ13, Cu-ACQ14, and Cu-ACQophen, were acquired by the Bruker AXS D8 Advance DaVinci diffractometer. Complexes Cu-ACQ13 and Cu-ACQophen presented as amorphous and Cu-AQC14 present more than one single phase. However, the crystal structure of Cu-ACQ12 was solved by PXRD analysis as in previous works.^41–46^ Initially, the powder of Cu-ACQ12 was gently ground in an agate mortar, and it was deposited in the hollow of a thin glass sample-holder plate, which has nearly zero background. Diffraction data were collected at RT, 293 ± 2 K, through overnight scans about 8 h, in 1 s per point in the 2θ range of 5–50° with a step of 0.02°, and it is equivalent to only one scan using 12.8 s per point. The D8 diffractometer is equipped with Ni-filtered CuKα radiation (λ = 1.5418 Å) and a Lynxeye linear position-sensitive detector. The following optics were set up: primary beam Soller slits (2.94°), fixed divergence slit (0.3°), and receiving slit of 5.68 mm. The X-ray generator was set to 40 kV and 40 mA. Approximate unit cell parameters were determined using the first 21 standard peaks and indexed through the single-value decomposition approach^47^ implemented in TOPAS.^48^ Space group *P-*1 was chosen, and the cell parameters were refined using the 4–50° (2θ) range by the Pawley method,^49^ giving *R*_wp_ = 0.0176. Then, the structure solution process was performed by the simulated annealing method^50^ and also implemented in TOPAS. In this case, the Copper­(II) ion was left free of translation. The ACQ12 ligand rigid body model, based on single-crystal data of similar ACQ compounds,^51^ was built using the Z-matrix formalism and also left free of rotation and translation parameters, as well as torsion angles, as described in Chart [Fig chart1]. In the same way, the nitrate ion and hydroxide ion were idealized as a Z-matrix formalism with free rotation and translation. In the last step, i.e., the refinement stage, carried out by the Rietveld method,^52^ optical, unit cell, and background parameters modeled by a Chebyshev polynomial function were refined. The rigid body descriptions introduced in the solution step were kept in the final refinement. Isotropic thermal parameters, Beq = 3.2 (Å^2^), were assigned to light atoms, while the copper­(II) ion was set up as Beq = 4.4 (Å^2^). The structure of Cu-ACQ12 was drawn using the OLEX2 program.^41^ The final Rietveld refinement plots are depicted in Figure S49.

**1 chart1:**
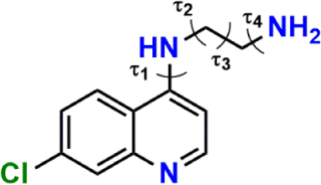
Sketch of ACQ12^a^

a The torsion angles refined in the PXRD analysis of Cu-ACQ12 are indicated as τ_n_. The hydrogens bonded to carbon atoms are omitted for clarity.

### 2.4 Antibacterial Assays

To evaluate the antimycobacterial activity, the standard drug rifampicin was used as an experimental control. The copper­(II) complexes and their respective ligands were solubilized in DMSO. The *Mtb* H37Rv strain (ATCC 27294) was prepared as a dense suspension of 10^5^ Colony Forming Units (CFU) mL^–1^. Subsequently, 100 μL of this suspension was dispensed into each well of a 96-well plate, excluding the wells reserved for the compound and culture medium controls. The compounds to be evaluated were added to a volume of 100 μL, and serial dilutions were performed. The plates were incubated for 7 days at 37 °C in 5% CO_2_ atmosphere. After the incubation time, 30 μL of 0.01% resazurin solution, previously dissolved in distilled water, was added to each well. The fluorescence intensity, indicative of bacterial growth, was measured after 24 h using a Biotek Synergy H1 plate reader. The MIC_90_ values, defined as the compound concentration that achieves 90% reduction in bacterial growth, were determined from the obtained data. The presented results represent the average of three independent assays performed under identical conditions.^53^

### 2.5 In Vitro Cytotoxic Activity Assays

Cytotoxic activity assays, half-maximal inhibitory concentration (IC_50_), were conducted following the methodology of Primo et al. Briefly, MRC-5 cells (diploid cell line of fibroblast) were cultured in Dulbecco’s Modified Eagle Medium (DMEM) (Vitrocell), supplemented with 10% fetal bovine serum, and fortified with 50 mg L^–1^ of gentamicin sulfate and 2 mg L^–1^ of amphotericin B. To ensure optimal growth and cellular propagation, MRC-5 cells were housed in culture flasks and maintained in a controlled environment at 37 °C with 5% CO_2_ until complete confluence was attained. Subsequently, a concentration of 2.5 × 10^5^ cells mL^–1^ of MRC-5 was prepared and allocated across 96 well plates, ensuring a consistent volume of 100 μL per well. After 24 h under the aforementioned conditions, proper cell adhesion was confirmed. Thereafter, compounds with concentrations ranging from 0.39 to 100 mg L^–1^ were introduced. In sequence, incubation stages were executed over intervals of 24, 48, and 72 h in a 5% CO_2_ atmosphere, using media devoid of antimicrobial and antifungal agents. Upon completion of the incubation process, each well was supplemented with 50 μL of 0.01% resazurin solution. After 3 h, fluorescence quantification was undertaken using the Synergy H1 instrument from Biotek. The selectivity index (SI) was subsequently calculated for each compound as the ratio of the IC_50_ value obtained in MRC-5 cells to the corresponding MIC_90_ value against *M. tuberculosis* H37Rv. Thus, the SI was calculated as the ratio IC_50_/MIC_90_ for each compound. The IC_50_ values were chosen as the cytotoxicity parameter because they represent a reproducible and time-dependent measurement of MRC-5 cell viability. While CC_50_ is often used as a general indicator of host-cell toxicity, the IC_50_ in MRC-5 cells was considered to be more relevant in the context of pulmonary tuberculosis, reflecting cytotoxicity in a clinically pertinent cell type.

### 2.6 DFT Studies

To investigate the interaction between Cu­(II) and the quinoline derivatives, density functional theory (DFT) calculations were performed.^54,55^ All molecular geometries were fully optimized at the energy minimum using the hybrid functional M06-2X^56^ in combination with the 6-31G basis set.^56–60^ Frequency calculations were carried out to ensure that the optimized structures corresponded to true minima on the potential energy surface; no imaginary frequencies were detected. Structural parameters were analyzed considering interatomic distances up to 3.00 Å.^61^ For the simulations of the electronic excited states, time-dependent density functional theory (TD-DFT) was employed using the M06-2X functional. Single-point calculations were performed on the optimized geometries using the 6-311G­(d,p) basis set for H, C, N, O, and Cl atoms and 6-31G for Cu. Ten singlet excited states were computed for each complex. The implicit Solvation Model Based on Density (SMD)^61^ was applied to simulate DMSO effects on the UV–vis absorption spectra. All calculations were performed using the Gaussian 16 software package (revision C.01).^62^ Molecular structures as well as the theoretical UV–vis and IR spectra were visualized and analyzed using GaussView 5.0.^63^

### 2.7 Synthesis of Cu­(II) Complexes with Quinoline Derivatives

#### 2.7.1 Synthesis of Cu-DCQ Complexes

About 1.00 mmol (0.242 g) of Cu­(NO_3_)_2_·3H_2_O, dissolved in 5 mL of CH_3_OH, was added dropwise to a solution containing 2.00 mmol (0.396 g) of DCQ in 10 mL of CH_3_OH. Then, the solution was kept under stirring for 10 min at RT, and purple polycrystalline material was formed. The precipitate was filtered off, washed three times with 10 mL of CH_3_OH:CH_3_CN (1:1), and dried in the air. Purple single crystals were obtained 1 day after part of the solid was solubilized in 30 mL of ethanol. Yield: 53%. Mp: 211 ± 1 °C. Elemental analysis (%) calcd for C_18_H_10_CuN_4_O_6_Cl_4_: C 37.04, H 1.73, N 7.60. Found: C 37.02, H 1.71, N 7.49. FT-IR (in cm^–1^): 1572 ν­(CC+CN); 1275 ν_asym_(NO_3_^–^); 1086 (νCCl.+δCH)_in plane_. FT-Raman (in cm^–1^): 1587 ν­(CC+CN); 1089 (νCCl.+δCH)_in plane_, 1041 ν_sym_(NO_3_^–^), 489 ν­(Cu–O), 206 ν­(Cu–N).

#### 2.7.2 Synthesis of Cu-ACQ Complexes

About 1.00 mmol (0.242 g) of Cu­(NO_3_)_2_·3H_2_O, dissolved in 5 mL of CH_3_OH:Et_2_O (1:1), was added to a solution containing the respective ACQ, 3.00 mmol, in 10 mL of CH_3_OH: Et_2_O (1:1), dropwise, and it was kept under stirring for 10 min at RT. Polycrystalline materials were obtained after 10 min. The precipitates were filtered off, washed three times with 10 mL of CH_3_OH, and dried in the air.

##### 2.7.2.1 Cu-ACQ12

Yield: 62%. Mp = 141 ± 1 °C. Elemental analysis (%) calcd for C_22_H_26_Cu_2_N_8_O_8_Cl_2_ (dinuclear): C 36.27, H 3.60, N 15.38. Found: C 36.49, H 3.85, N 15.97. FT-IR (in cm^–1^): 1593 ν­(CC+CN); 1334 ν_asym_(NO_3_^–^); 1258 ν­(R_2_NH); 1055 (νCCl+dCH)_in plane_; 646 ν­(Cu–OH). FT-Raman (in cm^–1^): 1585 ν­(CC+CN); 1257 ν­(R_2_NH); 1047 ν_sym_(NO_3_^–^); 495 ν­(Cu–N); 287 ν­(Cu–O).

##### 2.7.2.2 Cu-ACQ13

Yield: 43%. Mp = 155 ± 1 °C. Elemental analysis (%) calcd for (C_24_H_30_Cu_2_N_8_O_8_Cl_2_)·0.5H_2_O (dinuclear): C 37.65, H 4.08, N 14.64. Found: C 37.91, H 4.05, N 14.02. FT-IR (in cm^–1^): 1580 ν­(CC+CN); 1319 ν_asym_(NO_3_^–^); 1255 ν­(R_2_NH); 1055 (νCCl+δCH)_in plane_; 644 ν­(Cu–OH). FT-Raman (in cm^–1^): 1589 ν­(CC+CN); 1251 ν­(R_2_NH); 1066 (νCCl+δCH)_in plane_; 1043 ν_sym_(NO_3_^–^).

##### 2.7.2.3 Cu-ACQ14

Yield: 30%. Mp = 185 ± 1 °C. Elemental analysis (%) calcd for (C_26_H_36_Cu_2_N_8_O_8_Cl_2_)·H_2_O (dinuclear): C 38.81, H 4.76, N 13.93. Found: C 39.01, H 4.41, N 13.63. FT-IR (in cm^–1^): 1582 ν­(CC+C=N); 1334 ν_asym_(NO_3_^–^); 1269 ν­(R_2_NH); 1068 (νCCl+dCH)_in plane_; 645 ν­(Cu–OH). FT-Raman (in cm^–1^): 1587 ν­(CC+CN); 1268 ν­(R_2_NH); 1076 (νCCl+δCH)_in plane_; 1045 ν_sym_(NO_3_^–^); 503 ν­(Cu–N); 304 ν­(Cu–O).

##### 2.7.2.4 Cu-ACQophen

Yield: 64%. Mp = 206 ± 1 °C. Elemental analysis (%) calcd for C_30_H_25_CuN_7_O_4_Cl_2_: C 52.83, H 3.69, N 14.38. found: C 52.71, H 3.52, N 14.38. FT-IR (in cm^–1^): 1581 ν­(CC+CN); 1337 ν_asym_(NO_3_^–^); 1273 ν­(R_2_NH); 1055 (νCCl+δCH)_in plane_; 582 ν­(Cu–OH).

The general procedure for the synthesis of the five copper­(II) complexes is illustrated in Scheme 1.

**Scheme 1:**
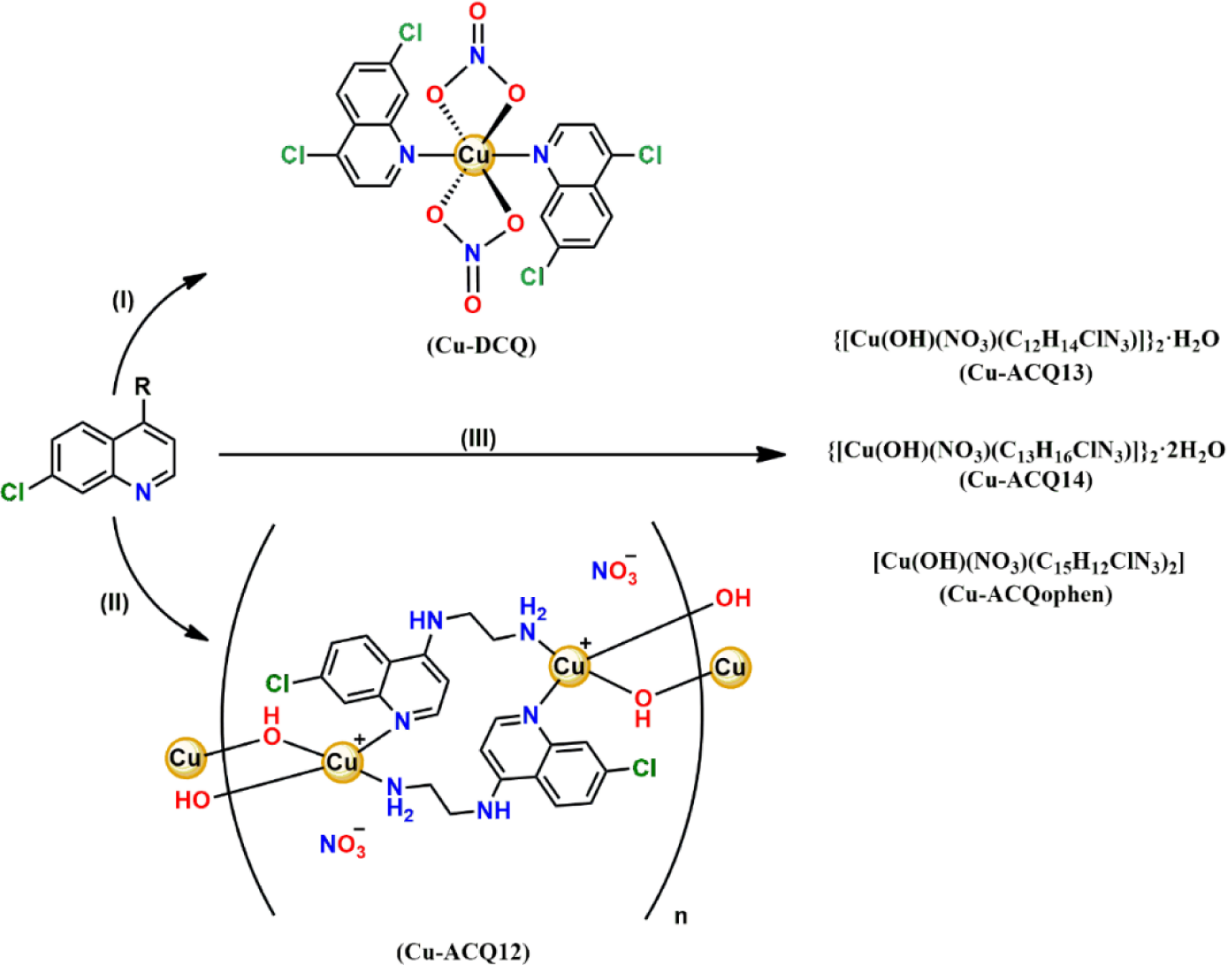
Syntheses of Copper­(II) Quinoline Derivative Complexes[Fn sch1-fn1]

a
**(I)** Cu­(NO_3_)_2_·3H_2_O, CH_3_OH, stirring, RT, 10 min; **(II)** Cu­(NO_3_)_2_·3H_2_O, CH_3_OH and Et_2_O, stirring, 10 min; **(III)** Cu­(NO_3_)_2_·3H_2_O, CH_3_OH and Et_2_O, stirring, 10 min.

## 3 Results and Discussion

### 3.1 IR and Raman Spectroscopy

Table 1 presents the possible assignments of the main observed signals in the FT-IR and FT-Raman spectra of copper complexes and their respective free ligands. All FT-IR and FT-Raman spectra for both the complexes and free ligands are provided as Figures S1–S11. FT-Raman measurements for Cu-ACQophen and the free ACQophen ligand were not feasible due to high fluorescence under Raman conditions. In the FT-IR spectra, the copper complexes exhibited shifts in the bands assigned to ν­(CC+CN) vibrations, associated with the breathing mode of the quinoline ring, within the 1593–1572 cm^–1^ range, suggesting coordination of the quinoline derivative to the Cu­(II) center through the nitrogen atom of the aromatic ring. Similarly, the ν­(CC+CN) band in the FT-Raman spectra of the complexes appeared in the 1589–1575 cm^–1^ range.^64,65^ Additionally, the copper complexes displayed strong bands between 1337 to 1275 cm^**–**1^ (FT-IR), assigned to ν_asym_(NO_3_^–^), and in the 1047–1020 cm^–1^ range (FT-Raman), corresponding to ν_sym_(NO_3_^–^). Particularly, for Cu-DCQ, the observed wavenumber shifts support coordination of nitrate to the Cu­(II) center (Section 3.4). The ν_asym_(NO_3_^–^) IR bands at 1275 cm^–1^ and the ν_sym_(NO_3_^–^) Raman at 1020 cm^–1^ suggest nitrate ion coordinated to the metal center.^66,67^ In contrast, for Cu-ACQ12, it is possible to find the ν_asym_(NO_3_^–^) at 1334 cm^–1^ and ν_sym_(NO_3_^–^) Raman signal at 1047 cm^–1^, which are consistent with noncoordinated nitrate ion. The remaining copper complexes follow the same noncoordinated nitrate pattern observed for Cu-ACQ12. The ν­(CuOH) stretching vibration observed near 646–582 cm^–1^ in the FT-IR spectra of Cu-ACQ complexes suggests the partial substitution of nitrate ions by hydroxide ones.^68–71^ Bands assigned to ν­(Cu–N) vibrations (503–489 cm^–1^) and ν­(Cu–O) vibrations (304–206 cm^–1^) were observed only in the FT-Raman spectra of Cu-DCQ, Cu-ACQ12, and Cu-ACQ14 complexes.^72,73^ These findings support the coordination of the quinoline ligands to the Cu­(II) center through the aromatic nitrogen in all cases, as well as the coordination of nitrate ions in Cu-DCQ and hydroxide ions in the Cu-ACQ systems. Experimental IR bands and DFT-calculated vibrational frequencies show a close correspondence. The small differences between the experimental and DFT-calculated vibrational frequencies are mainly due to the simplified theoretical conditions, which consider isolated molecules in the gas phase, while the experimental spectra reflect solid-state effects, such as intermolecular interactions and hydrogen bonding. Despite those minor variations, the overall agreement confirms that the DFT models describe the vibrational behavior of the Cu­(II) complexes. The complete DFT IR plots are available in the SI (Figures S53–S57).

**Table 1:** DFT-Calculated Vibrational Frequencies and Experimental IR and Raman Data for the Cu­(II) Complexes and Their Corresponding Free Ligands^a^ ^b^ ^c^

	ATR-FTIR/cm^**–**1^ (Exp)			FT-IR/cm^**–**1^ (DFT)				FT-Raman/cm^**–**1^			
Compound	ν_(CC+CN)_	ν_asym(NO3_-_)_	ν_(Cu–OH)_	ν_(CC+CN)_	ν_asym(NO3_-_)_	ν_(Cu–OH)_	ν_(Cu–N)_	ν_(CC+CN)_	ν_sym(NO3_-_)_	ν_(Cu–N)_	ν_(Cu–O)_
DCQ	1606	–	–	–	–	–	–	1610	–	–	–
**Cu-DCQ**	1572	1275	–	1574	1251	–	504	1575	1020	489	206
ACQ12	1580	–	–	–	–	–	–	1581	–	–	–
**Cu-ACQ12**	1593	1334	646	1567	1313	659	477	1585	1047	495	287
ACQ13	1583	–	–	–	–	–	–	1579	–	–	–
**Cu-ACQ13**	1580	1319	644	1567	1315	665	480	1589	1043	–	–
ACQ14	1578	–	–	–	–	–	–	1579	–	–	–
**Cu-ACQ14**	1582	1334	645	1586	1315	660	468	1587	1045	503	304
ACQophen	1565	–	–	–	–	–	–	–	–	–	–
**Cu-ACQophen**	1581	1337	582	1531	1274	604	508	–	–	–	–

a ν: stretching.

b ν_sym_: symmetric stretching.

c ν_asym_: asymmetric stretching.

### 3.2 UV–Vis Spectroscopy

The electronic spectra of the Cu­(II) complexes were recorded in DMSO solution at RT (293 ± 1 K), and the corresponding data are presented in Figure 3 and Table 2. The absorption maxima (λ_max_) and molar absorptivities (ε) reported in Table 2 correspond to the main bands observed in the experimental spectra, selected according to their relative intensities and spectral definition. The tentative assignments represent dominant transitions contributing to the observed absorption features, including the most intense π → π* transitions in the UV region and the lower-energy ligand-field (d–d) transitions observed between 400 and 850 nm. The absorption spectra of the ligands and their Cu­(II) complexes exhibit intense bands in the ultraviolet region, centered between 323 and 338 nm, which are assigned to intraligand π → π* transitions associated with the aromatic quinoline framework. Upon coordination to the metal center, significant changes in the spectral profiles are observed. A hyperchromic effect is noted for complexes Cu-DCQ, Cu-ACQ13, and Cu-ACQ14, indicating enhanced electronic conjugation upon coordination, whereas Cu-ACQ12 exhibits a hypochromic effect, suggesting slight electronic localization or decreased delocalization within the aromatic system. The Cu-ACQophen complex shows pronounced modification in the range 300–400 nm, consistent with the extended conjugation of the *o*-phenylenediamine moiety. In the visible region, low-intensity and broad bands are observed. The Cu-DCQ complex displays absorptions between 450 and 460 nm and 800–850 nm, which are typical of ligand-field (d–d) transitions for Cu­(II) centers in square-planar or distorted octahedral geometries.^74,75^ Similarly, the Cu-ACQ12, Cu-ACQ13, and Cu-ACQ14 complexes exhibit broad absorptions between 619 and 655 nm, consistent with square-pyramidal or distorted square-pyramidal coordination environments around the Cu­(II) ion.^76,77^ The qualitative assignments are supported by the energy ranges reported for analogous Cu­(II) systems in the literature.^74–77^ However, since the reported compounds are only similar to the described ones here, the comparison is only an approximate energetic correlation rather than a direct state-character correspondence. The UV–vis spectrum of Cu-ACQophen differs from the others, presenting one broad band with three well-defined shoulders between 460 and 600 nm, with molar absorptivity values typical of systems containing highly conjugated aromatic ligands.^78^ The calculated electronic absorption spectra (TD-DFT) were generated based on the DFT-optimized geometries, allowing qualitative comparison with the experimental results. Although molecular orbital analyses of the electronic transitions were not performed, the relative positions and intensities of the calculated bands showed good agreement with the experimental data, supporting the consistency of the proposed structural models. In addition, the combined DFT and molar conductivity data (Section 3.3) provided complementary support for the qualitative interpretation and experimental assignment of the absorption bands, reinforcing the coherence between the theoretical models and the spectroscopic behavior observed for the Cu­(II) complexes. All experimental UV–vis spectra and spectrophotometric titrations are provided in the SI (Figures S12–S21). The complete TD-DFT plots for Cu-ACQ12, Cu-ACQ13, and Cu-ACQ14 are also available in the SI (Figures S58–S60). The molar absorptivity, band position, and spectral profile of complexes remained unchanged over 24 h, indicating that the chromophoric species are stable in DMSO solution (SI, Figures S22–S30).

**Table 2:** Absorption Maxima (λ_max_), Molar Absorptivity (ε) Values and Possible Bands Assignments of Electronic Spectrometric Data for the Ligands and Cu­(II) Complexes Recorded in DMSO at RT (293 ± 1 K)^a^

Compound	λ_max_/nm (ε/M^–1^ cm^–1^)	Tentative Assignment
DCQ	323 (7299)^b^	π → π*, Intraligand transition
**Cu-DCQ**	323 (8425)^b^, 456 (11)^c^, 837 (26)^c^	π → π*, ligand-field (d–d) transitions
ACQ12	336 (18329)^b^	π → π* transition
**Cu-ACQ12**	337 (8973)^b^, 433 (278)^c^, 619 (150)^c^	π → π*, ligand-field (d–d) transitions
ACQ13	334 (16153)^b^	π → π* transition
**Cu-ACQ13**	336 (22248)^b^, 655 (108)^c^	π → π*, ligand-field (d–d) transition
ACQ14	335 (14449)^b^	π → π* transition
**Cu-ACQ14**	336 (17446)^b^, 628 (168)^c^	π → π*, ligand-field (d–d) transition
ACQophen	338 (23815)^b^	π → π* transition
**Cu-ACQophen**	325 (15613)^b^, 468 (11008)^b^, 498 (12797)^b^, 553 (9285)^b^, 597 (7485)^b^	π → π*, charge-transfer-type transitions

a ε = Molar absorptivity.

b (2.5 × 10^–5^ mol L^–1^, DMSO).

c (1.0 × 10^–3^ mol L^–1^, DMSO).

**Figure 3:**
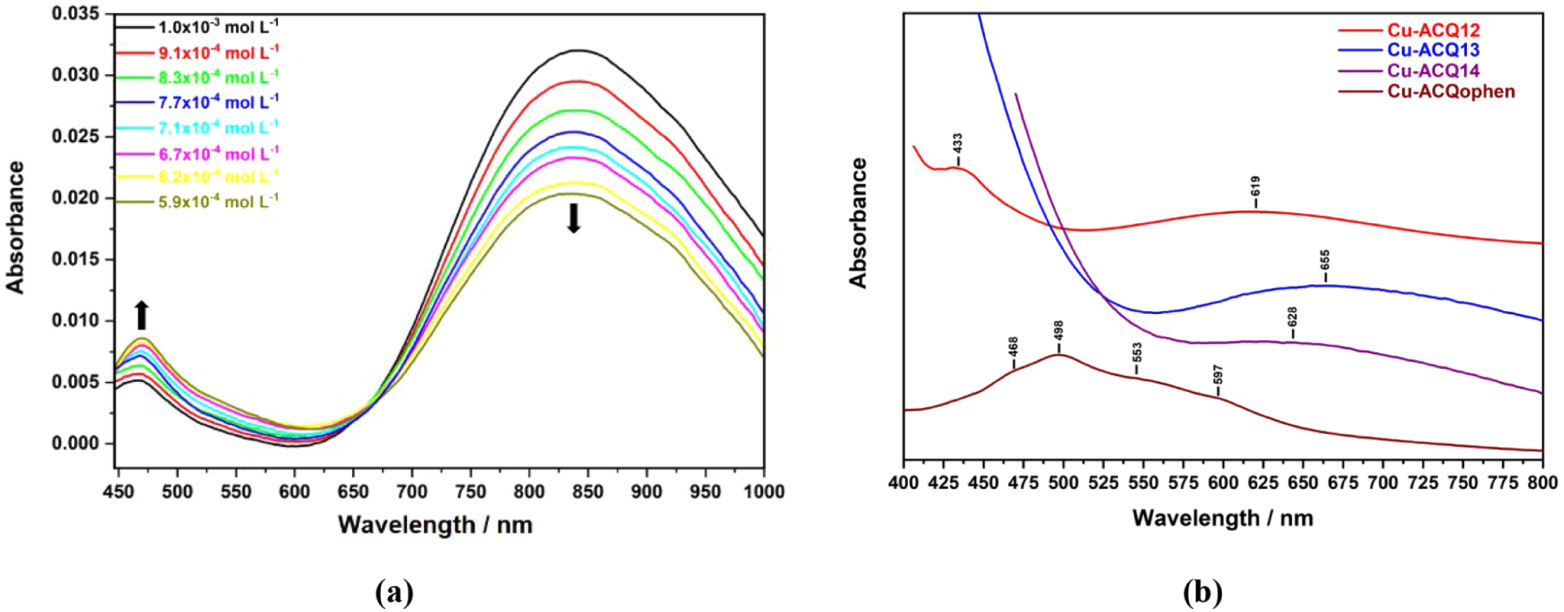
Spectrophotometric titrations for Cu-DCQ (a) and UV–vis absorption spectra of the obtained Cu-ACQ complexes (b).

### 3.3 Molar Conductivity Measurements

The molar conductivity measurements (μS cm^2^ mol^–1^) of the Cu-DCQ and Cu-ACQ complexes, summarized in Table 3, were performed in DMSO over time. Cu-DCQ showed the highest Λ_M_ value among the series, about 65 μS cm^2^ mol^–1^, which is near to 1:1 electrolyte, which is over 70 μS cm^2^ mol^–1^. The Cu-ACQ12 complex exhibits stable conductivity over 24 h, with Λ_M_ values that fall near the transition region typically observed between weakly dissociated species and nonelectrolytes in DMSO. For Cu-ACQ13 and Cu-ACQ14, a slight increase in conductivity is observed upon standing, but both complexes retain low Λ_M_ values characteristic of weakly dissociated systems. Cu-ACQophen also maintains low and time-independent conductivity, consistent with limited ionic dissociation.^79^ It is important to note that DMSO exhibits a well-known coordinating ability toward transition-metal centers. Therefore, coordination of DMSO to Cu­(II) ions in solution may be considered under the experimental conditions. This aspect represents an intrinsic limitation of conductivity measurements in DMSO, since partial solvent coordination may influence the extent of ion dissociation and the resulting molar conductivity values.

**Table 3:** Molar Conductivity Values, Λ_M_, of Copper­(II) Complexes—Cu-DCQ and Cu-ACQ System—in DMSO Solution (1 mm L^–1^) over Time (0 h, 2 h, 4 h, 6 h, and 24 h) at 298 ± 2 K^a^ ^b^

	Λ_M_ ± SD/μS cm^2^ mol^–1^				
	0 h	2 h	4 h	6 h	24 h
**Cu-DCQ**	65.1 ± 0.6	64.1 ± 0.6	63.4 ± 0.6	63.3 ± 0.6	60.1 ± 0.3
**Cu-ACQ12**	45.7 ± 0.1	47.7 ± 0.1	46.71 ± 0.07	46.4 ± 0.2	45.4 ± 0.1
**Cu-ACQ13**	20.5 ± 0.2	22.7 ± 0.7	24.1 ± 0.2	26 ± 1	39 ± 2
**Cu-ACQ14**	26 ± 1	29 ± 2	31 ± 3	35.8 ± 0.6	42.7 ± 0.6
**Cu-ACQophen**	26.1 ± 0.8	25.1 ± 0.8	25.3 ± 0.1	24 ± 1	25.9 ± 0.5

a (1.0 × 10^–3^ mol L^–1^, DMSO).

b SD = Standard Deviation.

### 3.4 Crystal and Theoretical Structures

The crystal structures of the Cu-DCQ and Cu-ACQ12 complexes are depicted in Figure 4. A summary of the main crystallographic parameters is provided in Table 4, and selected bond lengths and angles are presented in Table 5. Additional structural details, including intermolecular interactions, bond geometries, atomic coordinates, and molecular packing along the *a*, *b*, and *c* axes, are available in the SI (Figures S33–S48 and Tables S1–S8). The Cu-DCQ complex, Figure 4a, adopts a distorted octahedral geometry around the Cu­(II) center. The metal ion is coordinated by four oxygen atoms from two bidentate nitrate anions arranged in a *trans* chelating mode. The Cu–O bond lengths involving O1a and O1b are 1.978(9) and 2.006(9) Å, respectively, whereas the bonds to O2a and O2b are longer, measuring 2.486(9) and 2.485(9) Å; these distances are associated with *trans* oxygen pairs across the metal center. Additionally, two DCQ ligands coordinate to the Cu­(II) ion via the nitrogen atoms of their quinoline moieties, also in a *trans* configuration, with Cu–N bond distances of 2.004(9) and 2.006(9) Å. The linearity of the coordination environment is defined by the nearly collinear nitrate oxygen atoms (O1a–Cu–O1b angle of 179.5(5)°). The supramolecular organization of Cu-DCQ is stabilized by nonclassical hydrogen bonds of the C–H···Cl type, with a donor–acceptor (D···A) separation of 3.12(9) Å. The Cu-ACQ12 complex, Figure 4b, is characterized as an asymmetric μ-dihydroxido-bridged coordination polymer, where an inversion center located at the center of a parallelogram defines the [Cu­(μ–OH)]_2_ unit. In the asymmetric unit, the Cu­(II) center is coordinated by one hydroxide oxygen atom and one ACQ12 ligand via the nitrogen atom of its quinoline ring. The Cu–O bond lengths of 2.090(3) and 2.725(9) Å are consistent with those reported in similar systems.^80^ The polymeric structure propagates through successive Cu­(II) centers bridged by both the quinoline and the amino groups of the ACQ12 ligands. The coordination geometry around the copper center can be described as distorted square-planar, as evidenced by the X–Cu–X bond angles (X = N or O) ranging from 80.47(1)° to 97.86(7)° in the *cis* positions, and 147.35(9)° and 171.16(9)° in the *trans* arrangements. The angular deviations from the ideal square-planar values (90° and 180°) reflect the minor distortion of the coordination environment, commonly observed in Cu­(II) complexes due to Jahn–Teller effects and ligand-field asymmetry.^81,82^ A bridging Cu–O–Cu^ii^ angle of 82.13(3)° confirms the extended polymeric nature of the complex. Intramolecular stabilization is further achieved via C–H···O nonclassical hydrogen (C8–H8···O1), with a D···A distance of 3.15(2) Å. The supramolecular framework is reinforced by additional C–H···Cl nonclassical hydrogen (D···A = 3.69(3) Å) and π···Cl interactions with a centroid-Cl distance of 3.18(9) Å. Notably, moderate noncovalent interaction is observed between the nitrate ion and the Cu­(II) center, with a Cu···ONO_2_ contact of 2.89(8) Å.

**Figure 4:**
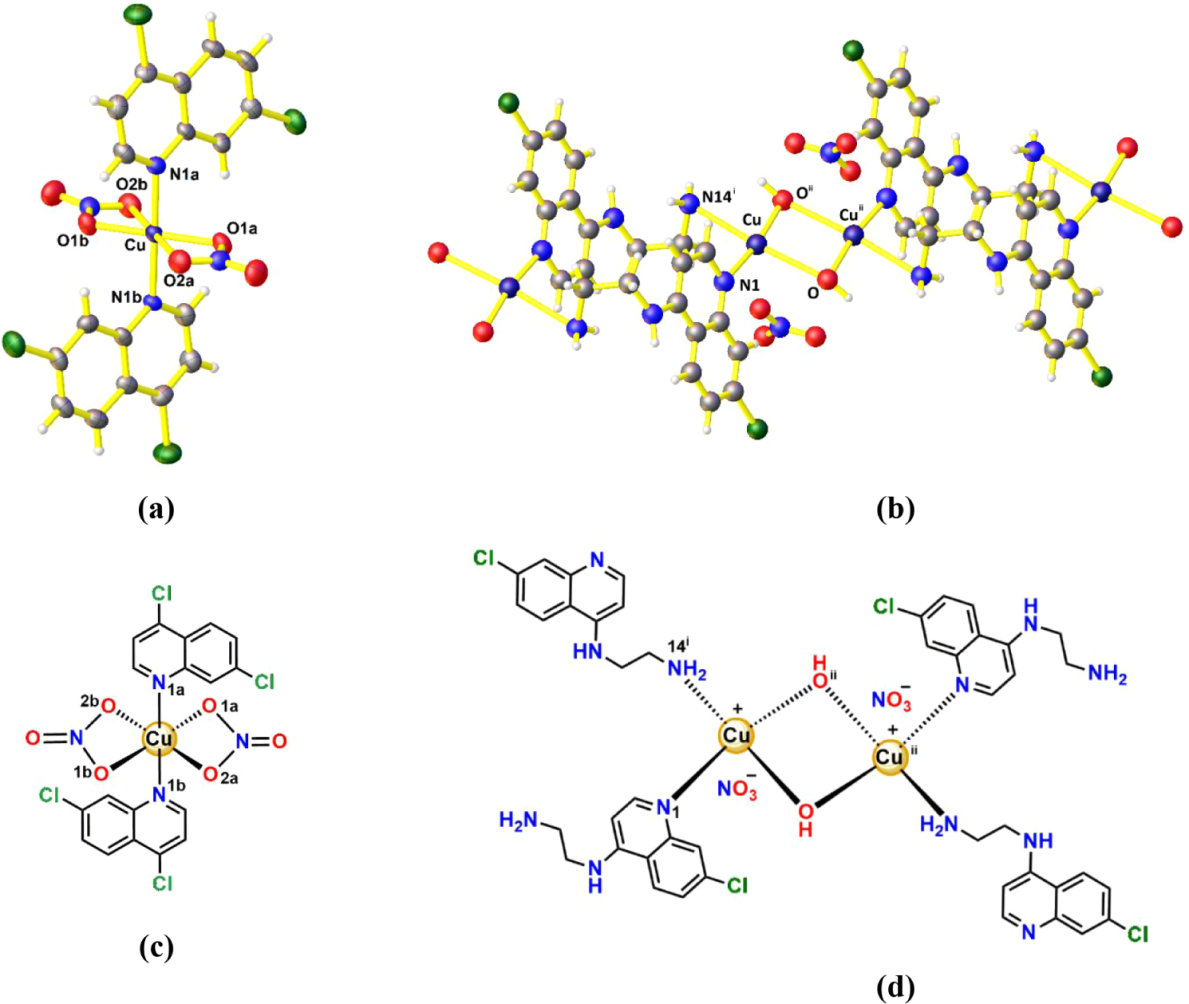
Crystal and schematic structures of Cu-DCQ (a,c) and Cu-ACQ12 (b,d), showing the coordination environments and donor atoms around the Cu­(II) centers. The drawn diagram includes both molecular representations and crystallographic illustrations to highlight the atomic arrangement and bonding features observed in the solid state. Thermal ellipsoids, for Cu-DCQ, are drawn at the 50% probability level; hydrogen atoms are represented as spheres with an arbitrary radius. Color codes: Cu, indigo; C, gray; H, white; O, red; N, blue; Cl, green.

**Table 4:** Crystallographic Data for Cu-DCQ and Cu-ACQ12^a^ ^b^

	Cu-DCQ	Cu-ACQ12
Diffractometer	Agilent SuperNova	Bruker AXS D8 Da Vinci
Scan type	ω	θ–θ
Empirical formula	C_18_H_10_Cl_4_CuN_4_O_6_	C_11_H_13_CuClN_4_O_4_
FW/g mol^–1^	583.64	364.24
*T*/K	293(2)	298
λ/Å	1.5418	1.5418
Crystal System	Orthorhombic	Triclinic
Space Group	*Pca2*_1_	*P-1*
a/Å	13.8551(3)	8.09(5)
b/Å	5.89080(10)	11.59(3)
c/Å	25.4729(4)	10.18(7)
α/deg	90	113.3(9)
β/deg	90	113.0(1)
γ/deg	90	75.7(3)
V/Å^3^	2079.04(7)	802.(9)
Z	4	2
*D*_*x*_/g cm^–3^	1.865	1.50(6)
Size/mm	0.613 × 0.088 × 0.17 Plate/deep blue	Polycrystalline green
Shape/Color		
F(000)	1164	370
μ/mm^–1^	6.651	26.766
2θ min – 2θ max/°	6.94–138.354	5–50
Measured reflections Independent reflections	15899	–
	3746 (*R*_int_ = 0.1181)	–
Reflections [*I > 2*σ*(I)*] Parameters/Restraints	3326	–
	298/1	48/–
Absorption correction	Multi-Scan	–
*T*_min_/*T*_max_	0.127/1.000	–
*R*_Bragg_/*R*_wp_	–	0.589/0.0246
*R*_1_/wR2 [*I > 2*σ*(I)*]	0.0966/0.2464	–
*R*_1_/wR2 (all data)	0.1011/0.2610	–
Goodness-of-fit on *F* ^2^	1.109	5.732
CCDC #	2434156	2434277

a **For complex Cu-DCQ:** Inferred from neighboring sites. H–atom parameters constrained. Least-squares matrix: full.

b **For complex Cu-ACQ12:** Hydrogen site location: rigid body. Aromatic hydrogen: (C–H distance = 0.95 Å, C–C–H = 120°). Aliphatic hydrogen: (C–H distance = 0.95 Å, C–C–H = 109.5°).

**Table 5:** Selected Bond Lengths and Angles for Cu-DCQ and Cu-ACQ12^a^

Complex	Lengths/Å		Angles/°	
**Cu-DCQ**			N1a–Cu–N1b	179.3(4)
			N1a–Cu–O1a	92.8(4)
	Cu–N1a	2.004(9)	N1a–Cu–O2a	93.5(3)
			N1a–Cu–O1b	87.7(4)
	Cu–N1b	2.006(9)	N1a–Cu–O2b	87.0(4)
			N1b–Cu–O1a	87.3(4)
	Cu–O1a	1.978(9)	N1b–Cu–O2a	87.1(4)
			N1b–Cu–O1b	92.2(4)
	Cu–O1b	2.006(9)	N1b–Cu–O2b	92.4(4)
			O1a–Cu–O2a	56.5(3)
	Cu–O2a	2.486(9)	O1a–Cu–O1b	179.5(5)
			O1a–Cu–O2b	123.9(3)
			O2a–Cu–O2b	179.3(4)
	Cu–O2b	2.485(9)	O1b–Cu–O2a	123.3(3)
			O1b–Cu–O2b	56.3(3)
**Cu-ACQ12**			N1–Cu–N14^i^	90.77(9)
	Cu–N1	1.990(8)	N1–Cu–O	90.73(1)
	Cu–N14^i^	2.668(7)	N1–Cu–O^ii^	171.16(9)
	Cu–O	2.725 (9)	N14^i^ – Cu–O^ii^	80.47(1)
	Cu–O^ii^	2.090(3)	N14^i^ – Cu–O	174.35(6)
	Cu···Cu ^ii^	3.199(9)	O–Cu–O^ii^	97.86(7)
			Cu–O–Cu^ii^	82.13(3)

a i = (1–*x*, 1–*y*, −*z*); ii = (1–*x*, 2–*y*, 1–*z*).

The molecular geometries of the complexes were optimized using Density Functional Theory (DFT) with the hybrid functional M06-2X and the 6-31G basis set in order to complement the crystallographic data and provide a deeper understanding of the coordination environment around the Cu­(II) center. The optimized structures reproduce the experimental features well, revealing distinct coordination numbers and geometries for each complex. Based on the analytical data, the complexes Cu-ACQ13 and Cu-ACQ14 have a molar proportion of 1:1 (M:L) while Cu-ACQophen crystallizes in a 1:2 (M:L) proportion. Complexes Cu-ACQ13 and Cu-ACQ14 exhibit a coordination number of four, with calculated Cu–L bond distances ranging from 1.78 to 2.38 Å, consistent with a distorted square-planar geometry typical of Cu­(II) systems with a d^9^ electronic configuration. The shorter Cu–O bonds (∼1.79 Å) correspond to strong equatorial interactions, whereas the slightly elongated Cu–N bonds (∼2.3 Å) indicate small deviations from planarity associated with Jahn–Teller distortions. The molar conductivity measurements in DMSO revealed very low conductivity values for Cu-ACQ13 and Cu-ACQ14, indicating nonelectrolytic behavior. This observation demonstrates that in solution, the complexes remain neutral molecular species without dissociation into ions. Such behavior provides valuable insight into their structural nature: in the solid state, both Cu-ACQ13 and Cu-ACQ14 behave as polymeric species, where Cu­(II) centers are interconnected through bridging ligands, forming extended coordination networks, similar to Cu-ACQ12. Upon dissolution in DMSO, partial disruption of the polymeric network occurs, allowing the nitrate ion to coordinate to the Cu­(II) center, thus completing the coordination sphere (NC = 4) as predicted by the DFT-optimized models. This process is consistent with the well-known lability of Cu­(II) complexes, which readily undergo coordination rearrangements in polar solvents to satisfy electronic and steric requirements.^83–85^ Overall, both the theoretical and experimental findings converge toward the same structural interpretation: polymeric species in the solid state, which transform into neutral species in the DMSO solution. In contrast, for the Cu-ACQophen complex, the proposed structural formulation is monomeric, consistent with the DFT-optimized geometry (NC = 5) and the calculated Cu–L bond lengths (Cu–O/N = 1.85–2.10 Å in the basal plane; elongated axial bond ≈ 2.39 Å), characteristic of a slightly elongated square-pyramidal geometry. In DMSO solution, the molar conductivity measurements exhibit very low values, indicative of nonelectrolytic behavior (≈25.28 S cm^2^ mol^–1^). This observation suggests that Cu-ACQophen remains neutral in solution, without dissociation into ionic species (1:1 or 1:2). Minor axial coordination rearrangements may occur upon dissolution, which is consistent with the moderate lability of Cu­(II) centers in polar media, yet without generating electrolyte species.^86,87^ The structural features are therefore compatible with a monomeric formulation that remains stable in aprotic, strongly coordinating solvents such as DMSO. The calculated vibrational frequencies show good agreement with the experimental IR spectra for all complexes. The main absorption bands are consistent with the coordination environments proposed from the DFT models, confirming that the structures remain stable upon coordination. The similarity between the experimental and theoretical profiles indicates that the computational approach accurately describes the structural and bonding features of the Cu­(II) complexes. Finally, it is possible to observe that the simulation results corroborate the experimental data for the formation of complex structures. The drawings of individual copper­(II) complexes as well DFT data are described in Tables S9 and S10.

### 3.5 Antibacterial Assays

MIC_90_ and IC_50_ biological assays were evaluated for synthesized copper complexes as well as for their respective free ligands. The biological outcomes bring evidence that Cu-ACQophen is a potential agent against mycobacterial infections, especially for *M. tuberculosis* H37Rv. The biological outcomes, some antitubercular coordination compounds, and some antitubercular drugs data are reported in Table 6.

**Table 6:** MIC_90_ and IC_50_ Values of Copper­(II) Complexes, Ligands, and Some Anti-*Mtb* Standard Drugs^a^ ^b^ ^c^ ^d^ ^e^ ^f^

Compounds	MW (g mol^–1^)	Metal (%)	MIC_90_ (μg mL^–1^ ± SD)	IC_50_ (μg mL^–1^ ± SD)	SI
DCQ^34^	198.05	–	4.6 ± 0.1	290 ± 5	63
**Cu-DCQ**	583.64	10.89	10 ± 3	128 ± 5	13
ACQ12 ^33^	221.69	–	10 ± 1	11.68 ± 1.9	1
**Cu-ACQ12**	728.48	17.45	3.1 ± 0.1	37 ± 2	12
ACQ13 ^33^	253.73	–	9 ± 1	12 ± 2	1
**Cu-ACQ13**	774.56	16.41	3.27 ± 0.02	26 ± 3	8
ACQ14 ^33^	285.77	–	2.4 ± 0.4	9.9 ± 1.65	4
**Cu-ACQ14**	820.63	15.49	3.0 ± 0.1	18 ± 2	6
ACQophen^33^	269.75	–	1.07 ± 0.01	208 ± 7	194
**Cu-ACQophen**	682.02	9.32	1.14 ± 0.02	55 ± 3	48
Cu(NO_3_)_2_·3H_2_O^27^	241.60	26.30	152 ± 38	–	–
[Cu(INH)(H_2_O)]SO_4_ 2H_2_O^88^	350.70	18.11	0.78*	–	–
[Cu(RIF)][Bibr ref89]	886.49	7.17	8 ± 4	–	–
[Cu(C_11_H_11_N_4_O_3_S)_2_(C_12_H_8_N_2_)]3H_2_O^27^	856.39	7.42	5320 ± 685	–	–
[Cu(C_2_H_4_NO_2_)(C_14_H_12_N_2_)(H_2_O)]NO_3_ ^90^	425.88	14.92	3.13*	–	–
Isoniazid (INH)^46^	137.06	–	<0.71*	–	–
Rifampicin (RIF)^46^	822.95	–	<0.012*	–	–
Ofloxacin^46^	361.37	–	1.05*	–	–
Amikacin^46^	585.61	–	0.96*	–	–
Estreptomycin^46^	581.57	–	0.47*	–	–
Moxifloxacin^46^	401.43	–	1.56*	–	–
Tobramycin^91^	467.47	–	8.56–17.10*	–	–
Clarithromycin^91^	747.96	–	10.70–21.40*	–	–
Cycloserine^91^	102.09	–	12.50–50 *	–	–

a IC_50_: half-maximal inhibitory concentration (MRC-5 cells).

b MW: Molar Weight.

c MIC_90_: lowest concentration of the antibiotic at which 90% of the isolates are inhibited (*M. tuberculosis* H37Rv).

d SD: Standard Deviation.

e SI: Selectivity Index (IC_50_ MIC_90_^–1^). SI values were derived by dividing the IC_50_ values obtained in MRC-5 cells by the MIC_90_ values against *Mtb* H37Rv. This approach allows a direct comparison of potency and host-cell safety, although it should be noted that the SI values reported here are based on IC_50_ rather than CC_50_ data

f *Standard deviation not reported by the author.

The biological behavior of free ligands as well as their respective copper­(II) complexes can be rationalized by considering both structural and electronic factors. The copper coordination sphere introduces redox activity (Cu­(II)/Cu­(I)) that is absent in the free ligands, which may account for the observed enhanced or differential biological effects. Also, steric and conformational constraints imposed by the chelation mode (e.g., octahedral or μ-hydroxido polymeric geometries) contribute to divergent physicochemical profiles. Indeed, recent literature emphasizes copper complexes as promising antimicrobial agents due to their intrinsic ability to generate reactive oxygen species (ROS), disrupt microbial membranes, and interfere with nucleic acids and metalloenzymes. In fact, the choice of the metal center can change the physicochemical properties, such as lipophilicity, chemical stability, geometry, and so on, and consequently, change the biological properties.^92^

The copper­(II) compounds evaluated in this study exhibited lower or equivalent MIC_90_ values when compared to standard anti-*Mtb* agents such as cycloserine, clarithromycin, tobramycin, moxifloxacin, amikacin, and ofloxacin. Yet, the MIC_90_ values of Cu-DCQ/ACQ are slightly higher of MIC_90_ values of the first-line drugs such as rifampicin (RIF), isoniazid (INH), and streptomycin. Nonetheless, the data of IC_50_ and consequently SI values for several anti-*Mtb* drugs are not available in the literature. Even for well-known copper­(II) complexes such as Casiopeínas-Cu­(II) and other bis­(diimine)-Cu­(II) complexes, the cytotoxicity assays in human fibroblasts have been not reported.^93^

ACQ-based complexes exhibit MIC_90_ values lower than or nearly equal to antitubercular Cu­(II) complexes reported, such as the phenanthroline-sulfamer complex, [Cu­(C_11_H_11_N_4_O_3_S)_2_(C_12_H_8_N_2_)] 3H_2_O, the amino acidate-phenanthroline system CasIIgly ([Cu­(C_2_H_4_NO_2_)­(C_14_H_12_N_2_)­(H_2_O)]­NO_3_), and the classical first-line drugs INH and RIF in [Cu­(INH)­(H_2_O)]­SO_4_·2H_2_O and [Cu­(RIF)], respectively^24,94,95^ Overall, these comparisons demonstrate that the ACQ scaffold, particularly in the *o*-phenylenediamine derivative, provides Cu­(II) complexes with antimicrobial activity equivalent to systems constructed from ligands with intrinsically recognized antibacterial activity, underscoring their potential as promising metallodrug candidates.

Adjusting the length and polarity of aliphatic chains can significantly influence both activity and selectivity as suggested by several studies.^96–98^ In this way, among the described compounds, DCQ and Cu-DCQ, Cu-ACQ12 and Cu-ACQophen exhibited both potent antimicrobial activity and high selectivity. Specifically, the Cu-ACQophen complex was selected as the most promising candidate as an antitubercular drug since it displayed a higher selectivity index (SI ≈ 48) compared to the other active analogues (Cu-ACQ13, SI ≈ 8; Cu-ACQ14, SI ≈ 6; ACQ14, SI ≈ 4). According to previous studies, a selectivity index (SI) greater than 10 is considered of high selectivity and favorable therapeutic window.^99^ Similarly, antifungal compounds with SI = 16–32 were considered for *in vivo* studies,^100^ and antimicrobial peptides with SI near 10 were evaluated for preclinical tests.^101,102^

Previous studies have also demonstrated that 8-hydroxyquinoline derivatives display rapid bactericidal activity against *Mtb*, with efficacy strongly dependent on the presence of exogenous copper. Acting as copper ionophores, they elevate intracellular copper levels, leading to potent growth inhibition (MIC ≈ 0.16 μg/mL in the presence of Cu).^103^ The Cu­(II)-quinoline complexes reported here exhibit coordination geometries similar to those of Cu­(II)-8-hydroxyquinoline derivatives; as a result, the Cu-DCQ and Cu-ACQ retain low MIC_90_ and good IC_50_ values. The Cu-DCQ and Cu-ACQ12 compounds also can be classified as high selectivity compounds since their SI values are greater than 10. The complexes Cu-ACQ13 and Cu-ACQ14, despite the moderate SI (∼8) and (∼6), show MIC_90_ < 4.3 mg L^–1^. As commented on above, the decrease in selectivity may be associated with differences in physicochemical properties, influenced by an increase in the aliphatic chain.

## 4 Conclusions

Five novel Cu­(II) complexes containing quinolone derivative compounds were successfully synthesized and characterized by spectroscopic (IR, Raman, and UV–vis) and analytical techniques, including elemental analysis, conductivity, and X-ray diffraction. The crystal structures of Cu-DCQ and Cu-ACQ12 were determined by single-crystal and powder X-ray diffraction studies, respectively, revealing that the Cu­(II) centers adopt distorted octahedral geometry in Cu-DCQ, whereas a distorted square-planar environment is observed for Cu-ACQ12. Consistent with these findings, Cu-DCQ forms mononuclear species containing two coordinated DCQ ligands, while Cu-ACQ12 generates a polymeric Cu­(II) arrangement in which the metal centers are interconnected by μ–OH bridges. The structures of Cu-ACQ13, Cu-ACQ14, and Cu-ACQophen were established through DFT optimization. The calculated geometries indicate that Cu-ACQ13 and Cu-ACQ14 also adopt polymeric μ–OH-bridged architectures, whereas Cu-ACQophen corresponds to mononuclear species. DFT calculations provided complementary insights, corroborating the experimental findings and revealing distinct coordination geometries, such as distorted square-planar for Cu-ACQ13 and Cu-ACQ14, and slightly elongated square-pyramidal for Cu-ACQophen. The Cu­(II) center introduces redox activity (Cu­(II)/Cu­(I)) and geometrical rigidity that enhance antimicrobial behavior relative to the free ligands. All complexes displayed notable activity against *Mtb*, with MIC_90_ values comparable to or lower than those of several standard anti-*Mtb* agents. Among the described compounds, Cu-ACQophen exhibited the highest activity (MIC_90_ = 1.68 μmol L^–1^) and selectivity (SI ≈ 48), exceeding the SI > 10 threshold for high therapeutic potential. Cu-DCQ and Cu-ACQ12 also showed favorable selectivity (SI > 10), while Cu-ACQ13 and Cu-ACQ14 showed MIC_90_ < 4.3 mg L^–1^ despite the moderate SI values. These results demonstrate the structure–activity relationship, where coordination geometry and ligand substitution modulate both potency and selectivity. Overall, the Cu­(II)-quinoline systems represent promising scaffolds for the development of new antitubercular agents.

## Supplementary Material



## Data Availability

The data that support the findings of this study are available in the Supporting Information.

## Notes

ATR-FTIR and FT-Raman spectra for all complexes; UV–vis spectra for all complexes; ^1^H NMR spectra for ACQ ligands; crystallographic data for complexes Cu-DCQ and Cu-ACQ12; TD-DFT tables and spectra (PDF)
