# Supplementary material for: Selective Copper(II) Complexes against Mycobacterium tuberculosis
Source: ACS Omega. 2025 Dec 29;11(1):2114–27. doi: 10.1021/acsomega.5c10934 (PMC12809329; doi:10.1021/acsomega.5c10934)
Supplement: Supplementary file 1 [file ao5c10934_si_001.pdf]

Supporting Information for:

Selective Copper (II) complexes against *Mycobacterium tuberculosis*

Kaíque A. D'Oliveira [a], Nícolas Glanzmann [b], Adilson D. da Silva [b], Carlos E. T. Bruzeguini [c], Marcos A. Ribeiro [c], Christian S. C. Canales [d,e], Cesar A. Roque–Borda [d], Fernando R. Pavan [d], Débora F. M. da Silva [f], Douglas H. Pereira [f], and Alexandre Cuin[a*]

[a] Laboratório de Química Bioinorgânica, Department of Chemistry, Institute of Exact Sciences, Federal University of Juiz de Fora (UFJF), Juiz de Fora, Minas Gerais 36036–330, Brazil

[b] Department of Chemistry, Institute of Exact Sciences, Federal University of Juiz de Fora (UFJF), Juiz de Fora, Minas Gerais 36036–330, Brazil

[c] Department of Chemistry, Institute of Exact Sciences, Federal University of Espírito Santo (UFES), Vitória, ES 29075–910, Brazil

[d] School of Pharmaceutical Sciences, Department of Biological Sciences, São Paulo State University (UNESP), Araraquara, SP 14800–900, Brazil

[e] School of Pharmacy, biochemistry and biotechnology, Santa Maria Catholic University, Arequipa 04013, Perú

[f] Technological Institute of Aeronautics (ITA), São José dos Campos, São Paulo 12228–900, Brazil

*Email: alexandre.cuin@ufjf.br

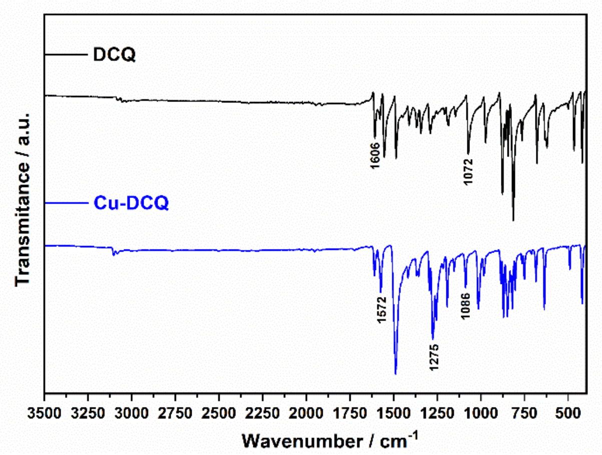

Figure S1: Experimental FT-IR spectra for complex Cu-DCQ and DCQ ligand

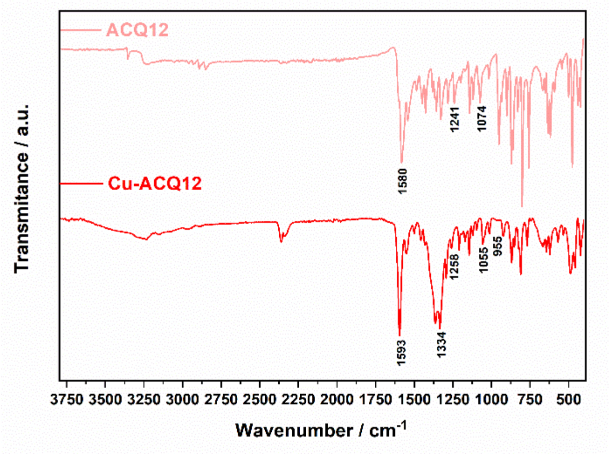

Figure S2: Experimental FT-IR spectra for complex Cu-ACQ12 and ACQ12 ligand

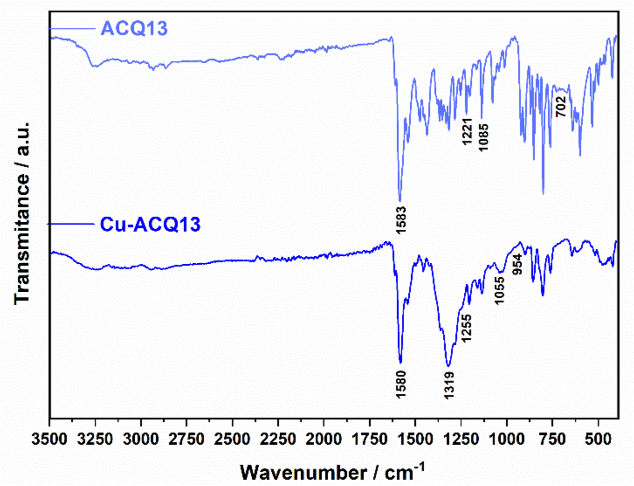

Figure S3: Experimental FT-IR spectra for complex Cu-ACQ13 and ACQ13 ligand

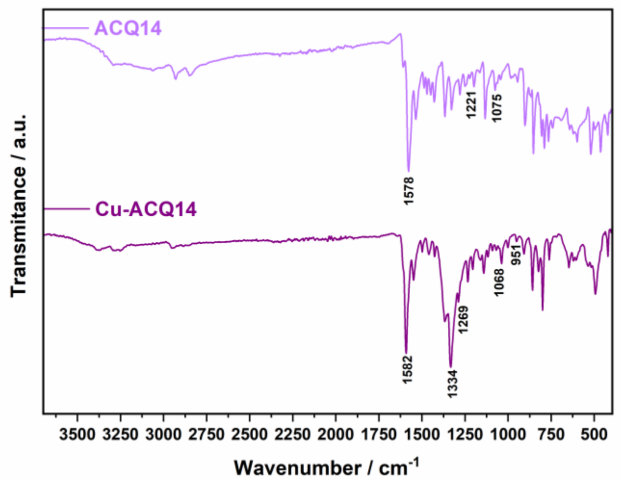

Figure S4: Experimental FT-IR spectra for complex Cu-ACQ14 and ACQ14 ligand

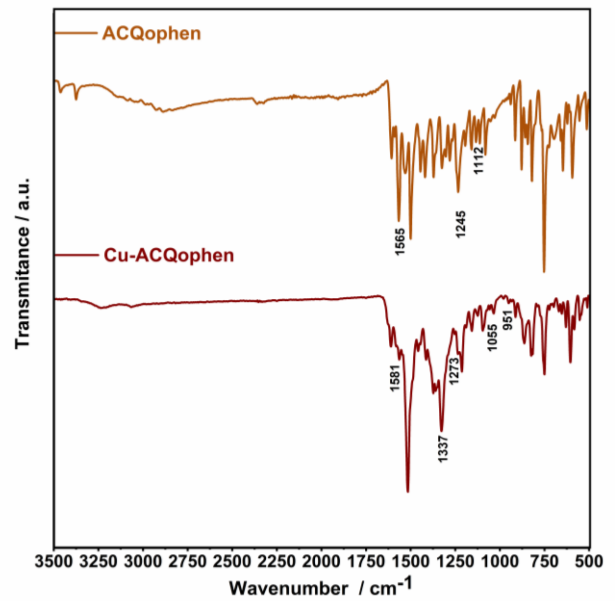

Figure S5: Experimental FT-IR spectra for complex Cu-ACQophen and ACQophen ligand

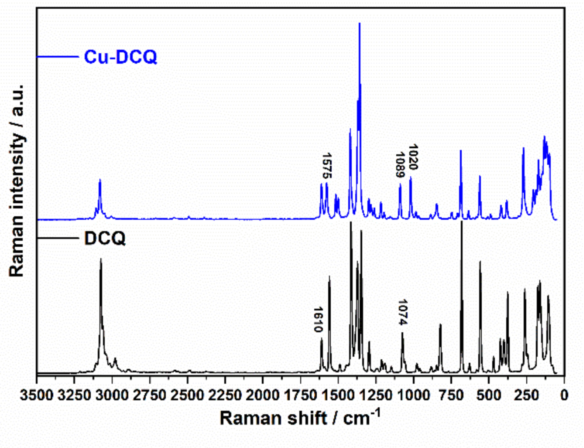

Figure S6. Experimental FT-Raman spectra for complex Cu-DCQ and DCQ ligand

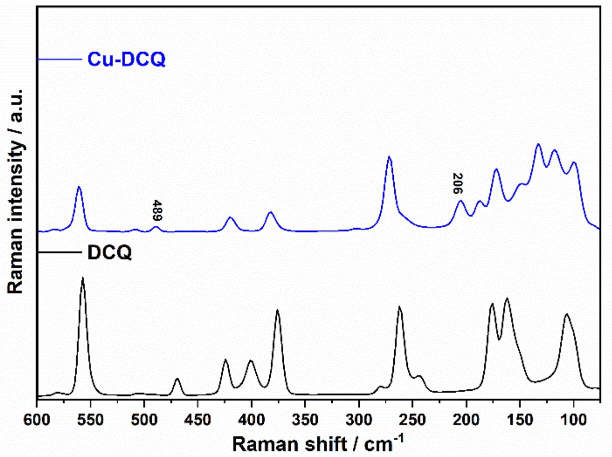

Figure S7. Experimental FT-Raman spectra for complex Cu-DCQ and DCQ ligand, range 600-50 cm^{-1}

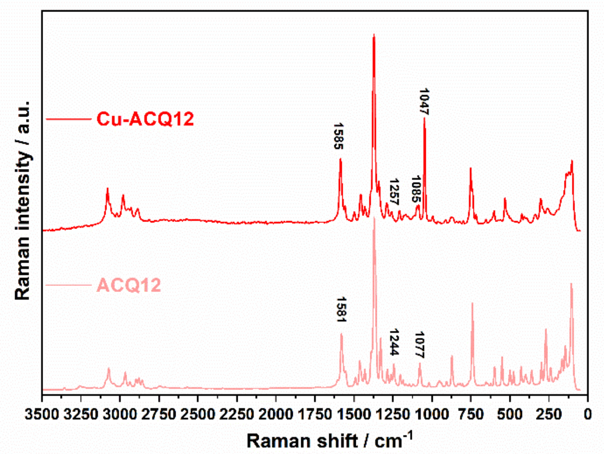

Figure S8. Experimental FT-Raman spectra for complex Cu-ACQ12 and ACQ12 ligand

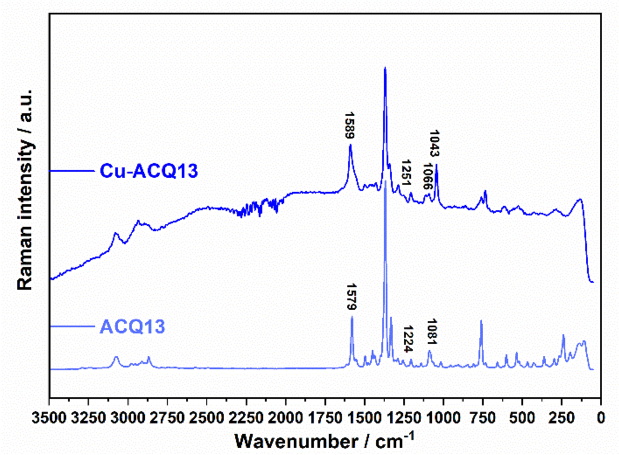

Figure S9. Experimental FT-Raman spectra for complex Cu-ACQ13 and ACQ13 ligand

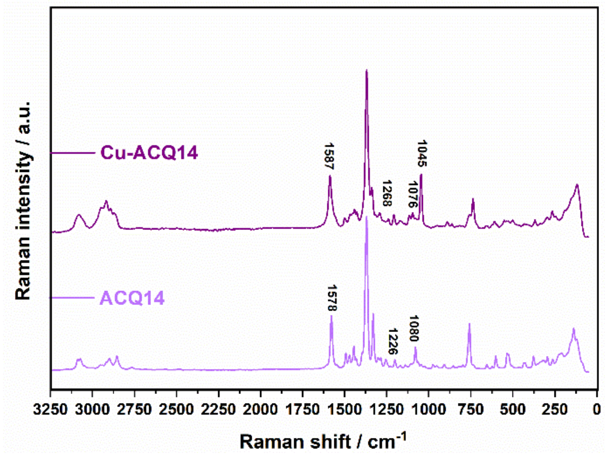

Figure S10. Experimental FT-Raman spectra for complex Cu-ACQ14 and ACQ14 ligand

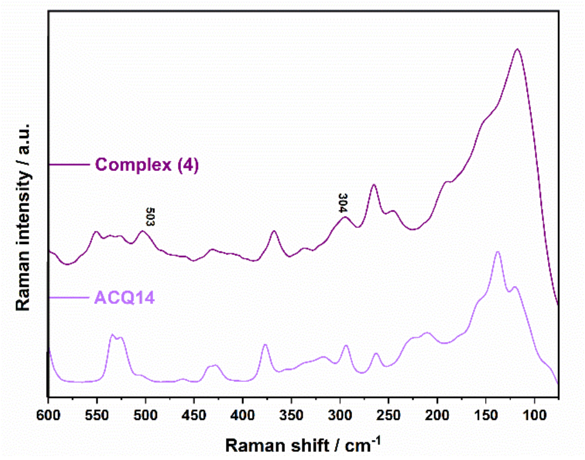

Figure S11. Experimental FT-Raman spectra for complex Cu-ACQ14 and ACQ14 ligand, range 600-50 cm^{-1}

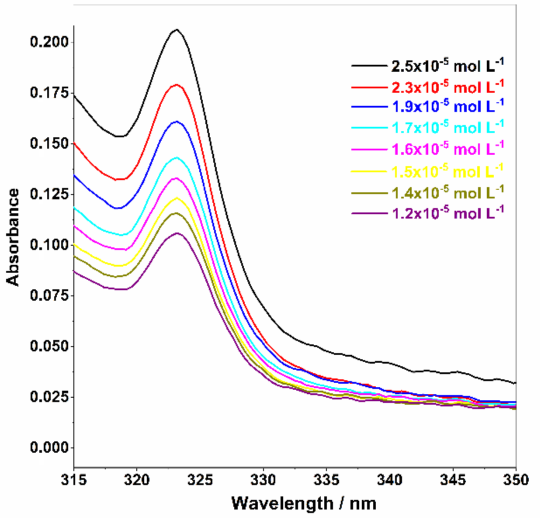

Figure S12. Spectrophotometric titrations and experimental UV-VIS spectra for complex Cu-DCQ, DMSO

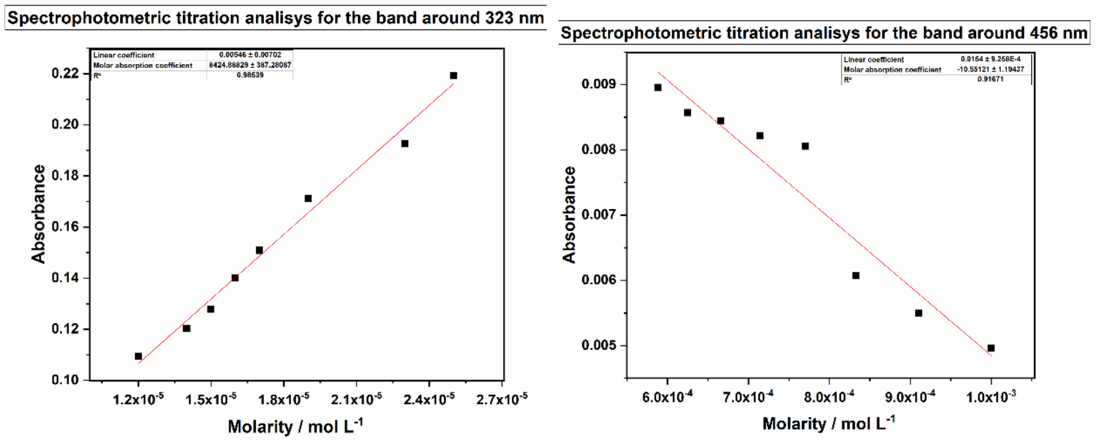

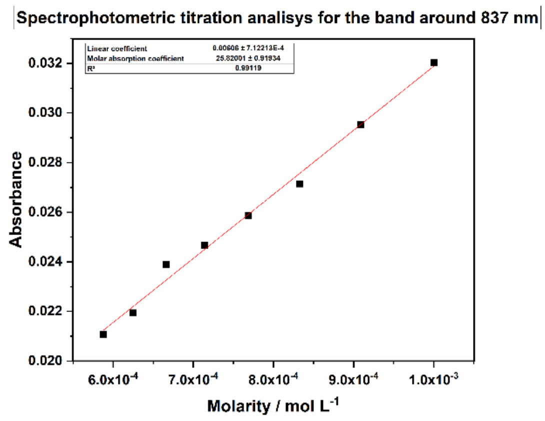

Figure S13. Spectrophotometric titrations analysis for complex Cu-DCQ

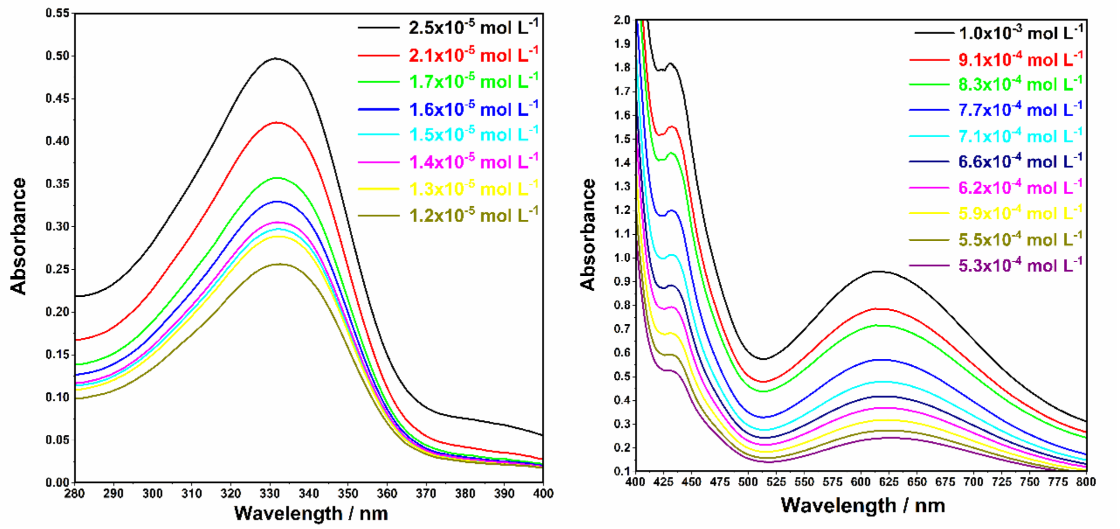

Figure S14. Spectrophotometric titrations and experimental UV-VIS spectra for complex Cu-ACQ12, DMSO

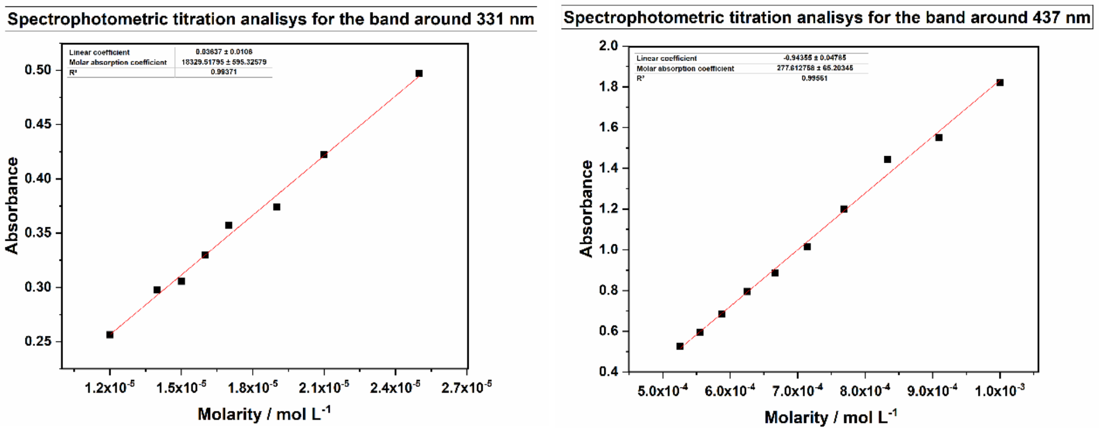

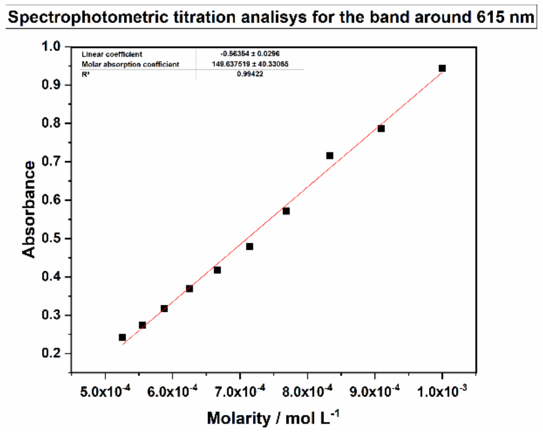

Figure S15. Spectrophotometric titrations analysis for complex Cu-ACQ12

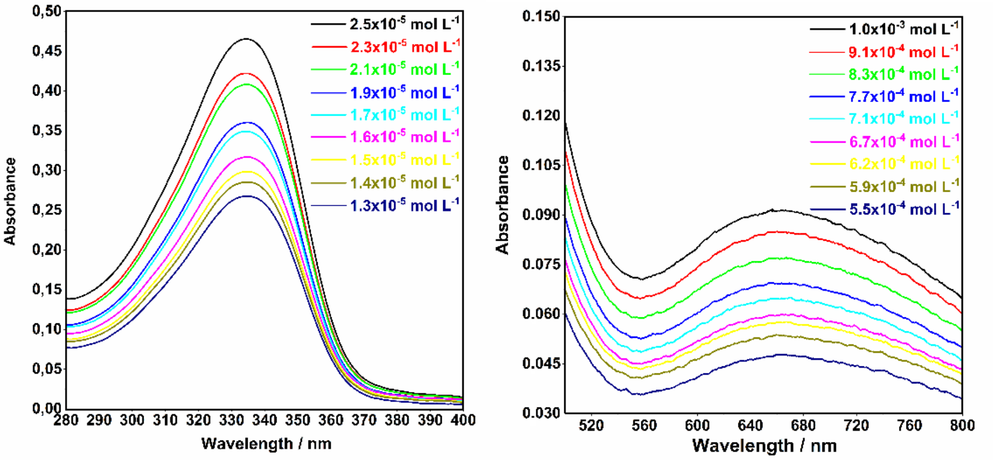

Figure S16. Spectrophotometric titrations and experimental UV-VIS spectra for complex Cu-ACQ13, DMSO

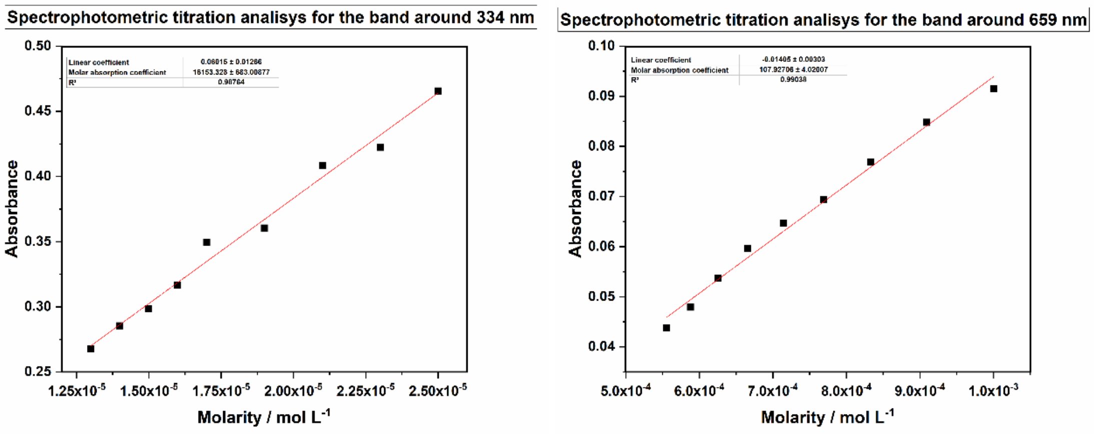

Figure S17. Spectrophotometric titrations analysis for complex Cu-ACQ13

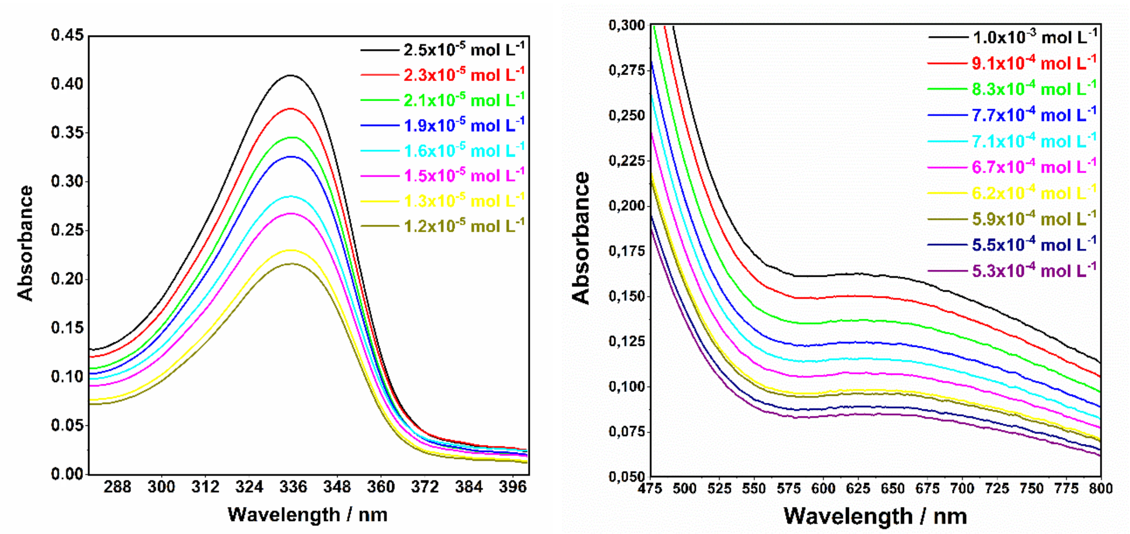

Figure S18. Spectrophotometric titrations and experimental UV-VIS spectra for complex Cu-ACQ14, DMSO

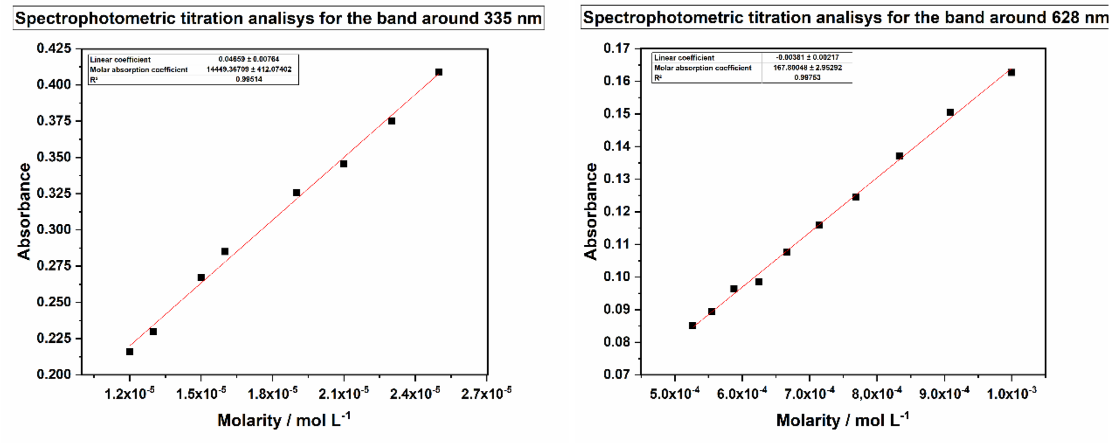

Figure S19. Spectrophotometric titrations analysis for complex Cu-ACQ14

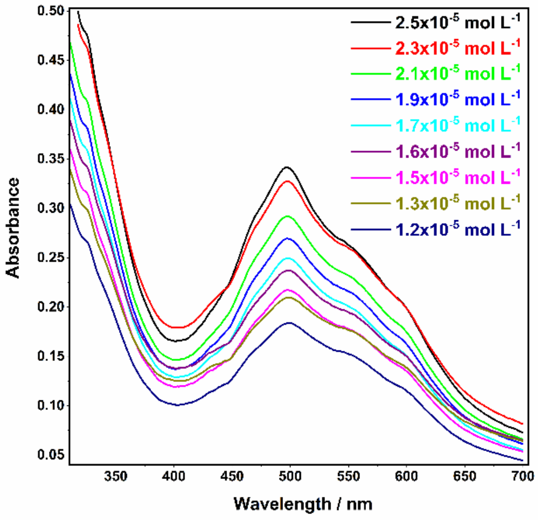

Figure S20. Spectrophotometric titrations and experimental UV-VIS spectra for complex Cu-ACQophen, DMSO

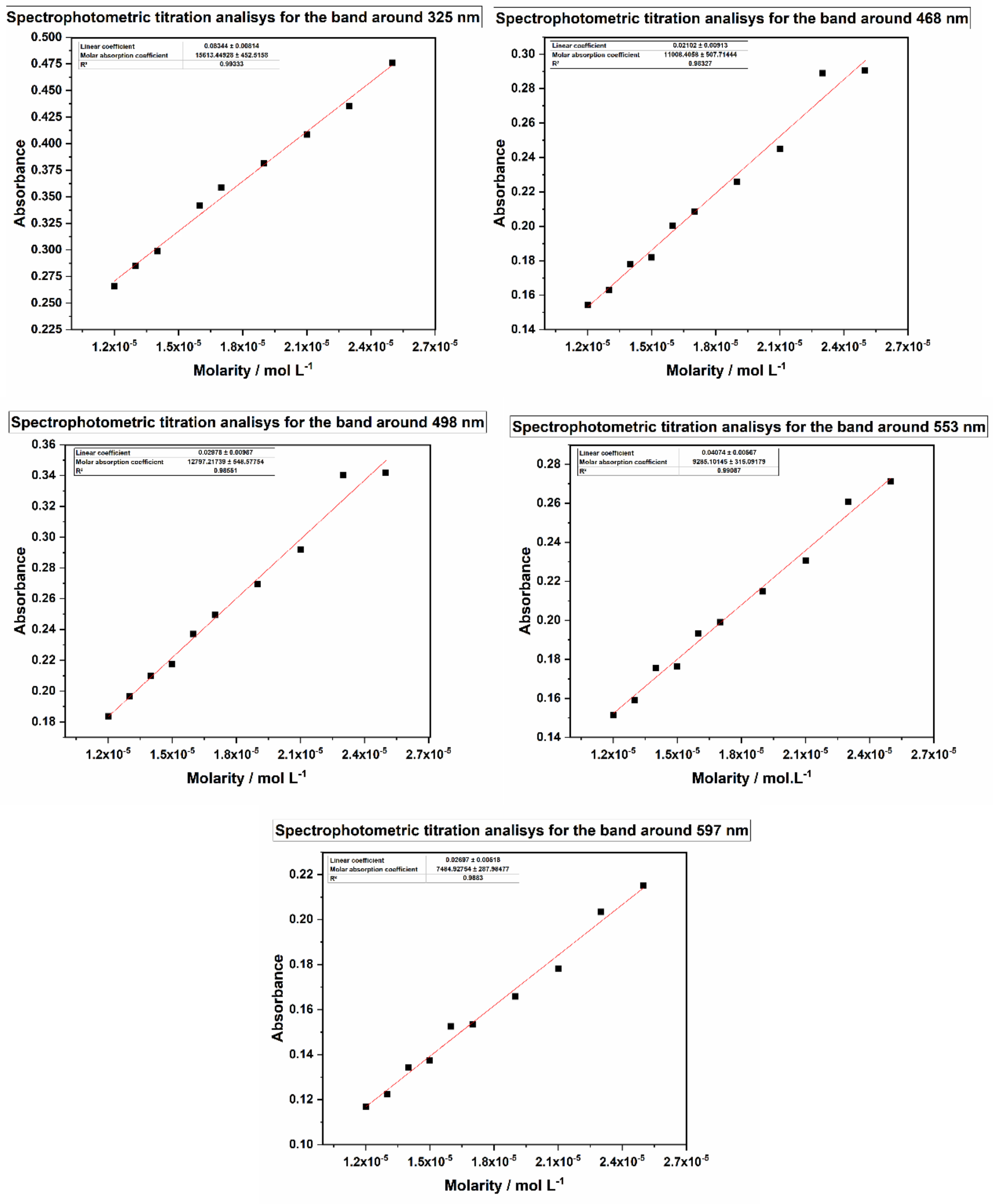

Figure S21. Spectrophotometric titrations analysis for complex Cu-ACQophen

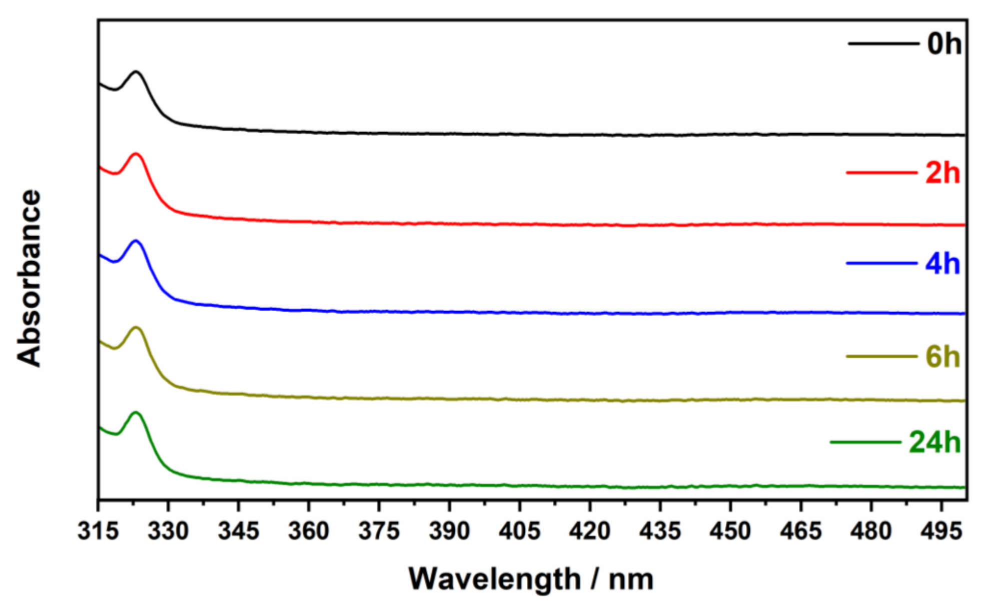

Figure S22. Experimental UV-VIS spectra over time for complex Cu-DCQ
Range: 315-500 nm (293 ± 1 K, 2.5x10^{-5} mol L^{-1}, DMSO)

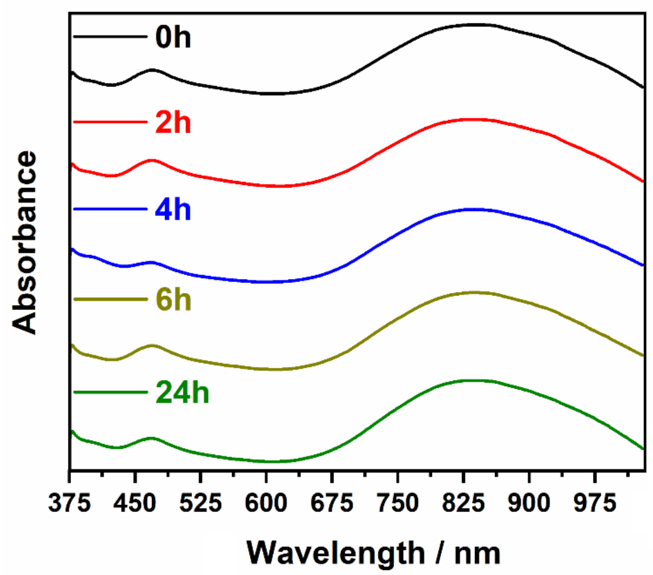

Figure S23. Experimental UV-VIS spectra over time for complex Cu-DCQ
Range: 375-1030 nm (293 ± 1 K, 1.0x10^{-3} mol L^{-1}, DMSO)

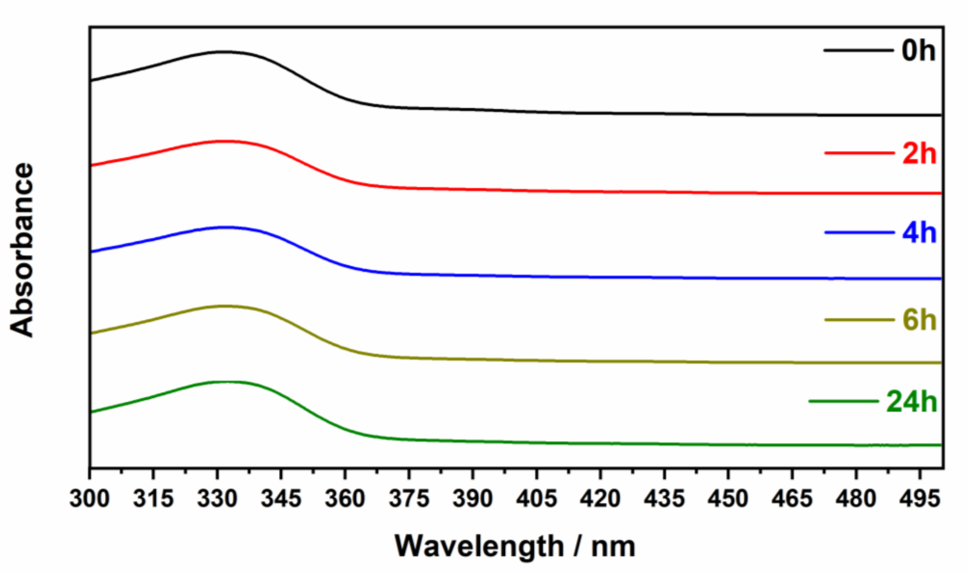

Figure S24. Experimental UV-VIS spectra over time for complex Cu-ACQ12
Range: 300-500 nm (293 ± 1 K, 2.5x10^{-5} mol L^{-1}, DMSO)

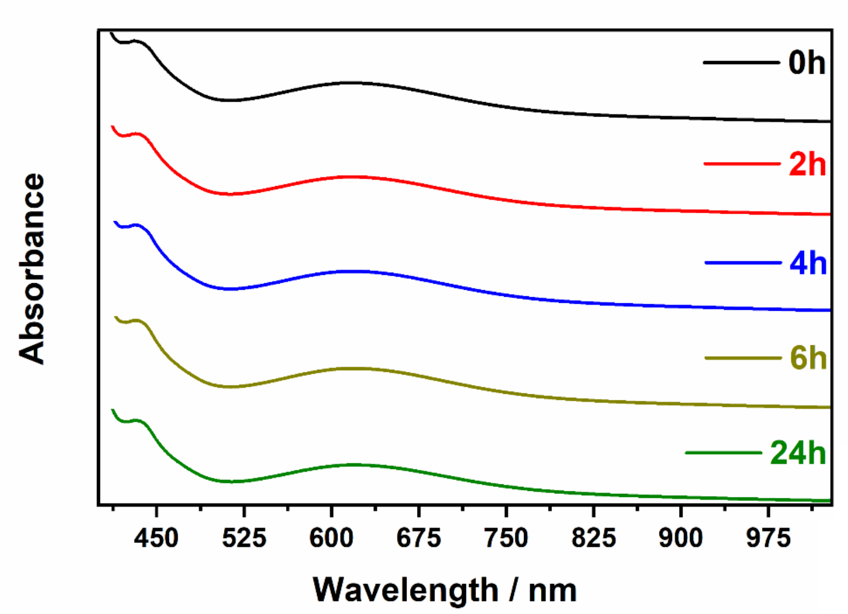

Figure S25. Experimental UV-VIS spectra over time for complex Cu-ACQ12
Range: 410-1030 nm (293 ± 1 K, 1.0x10^{-3} mol L^{-1}, DMSO)

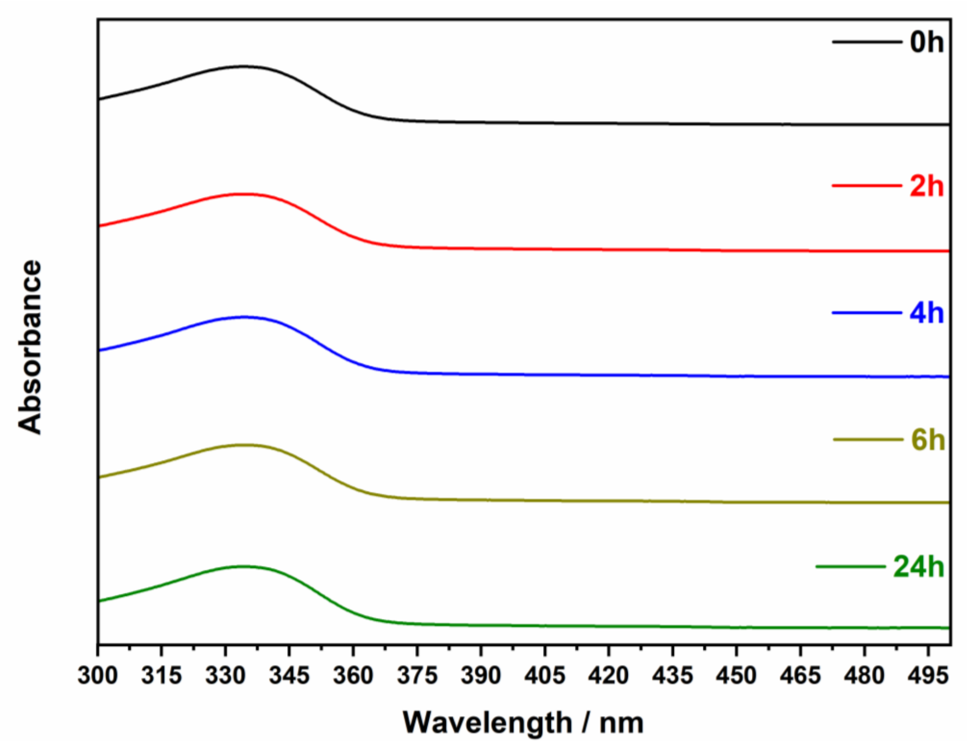

Figure S26. Experimental UV-VIS spectra over time for complex Cu-ACQ13
Range: 300-500 nm (293 ± 1 K, 2.5x10^{-5} mol L^{-1}, DMSO)

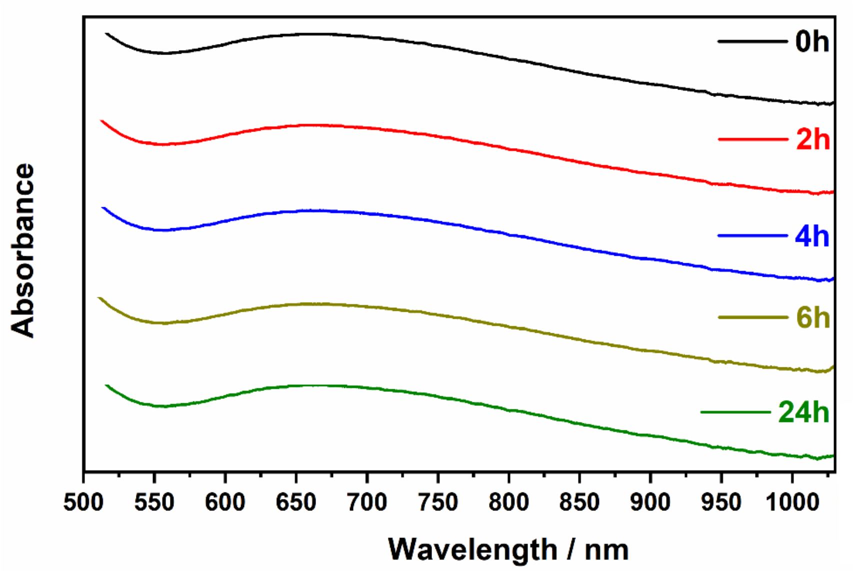

Figure S27. Experimental UV-VIS spectra over time for complex Cu-ACQ13
Range: 410-1030 nm (293 ± 1 K, 1.0x10^{-3} mol L^{-1}, DMSO)

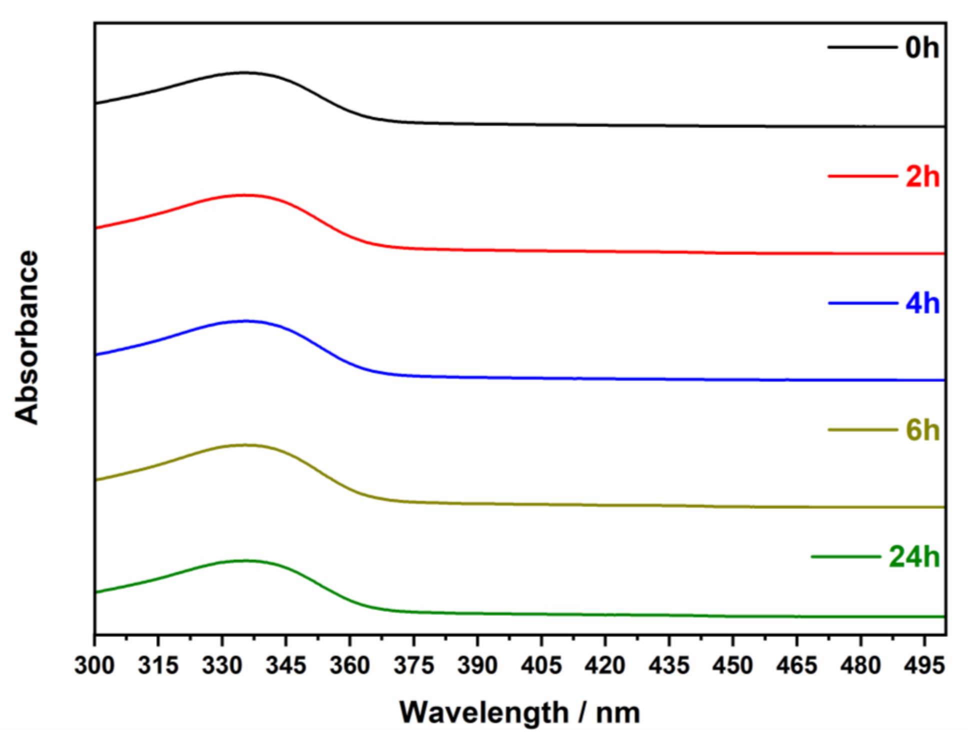

Figure S28. Experimental UV-VIS spectra over time for complex Cu-ACQ14
Range: 300-500 nm (293 ± 1 K, 2.5x10^{-5} mol L^{-1}, DMSO)

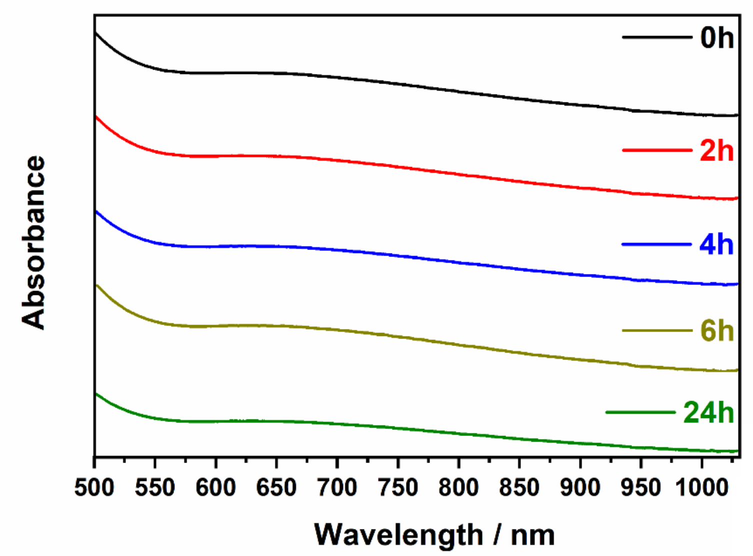

Figure S29. Experimental UV-VIS spectra over time for complex Cu-ACQ14
Range: 410-1030 nm (293 ± 1 K, 1.0x10^{-3} mol L^{-1}, DMSO)

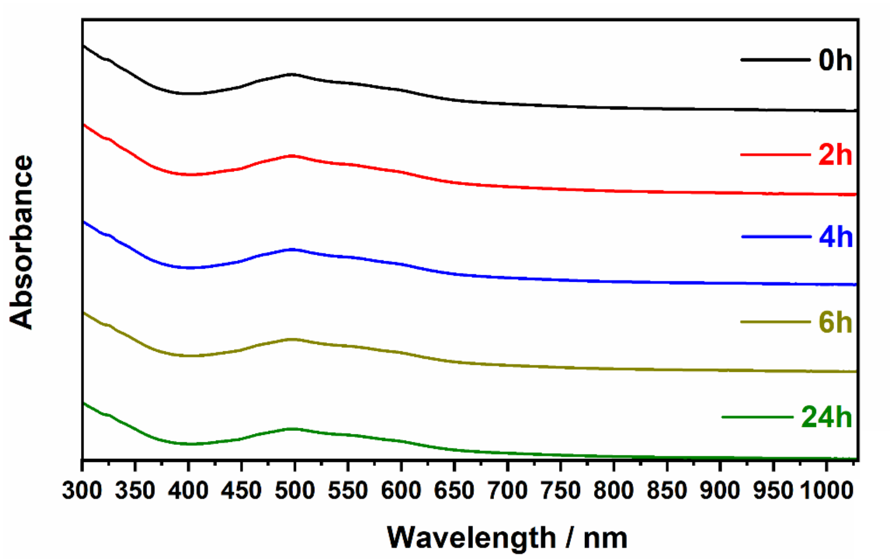

Figure S30. Experimental UV-VIS spectra over time for complex Cu-ACQophen
Range: 300-500 nm (293 ± 1 K, 2.5x10^{-5} mol L^{-1}, DMSO)

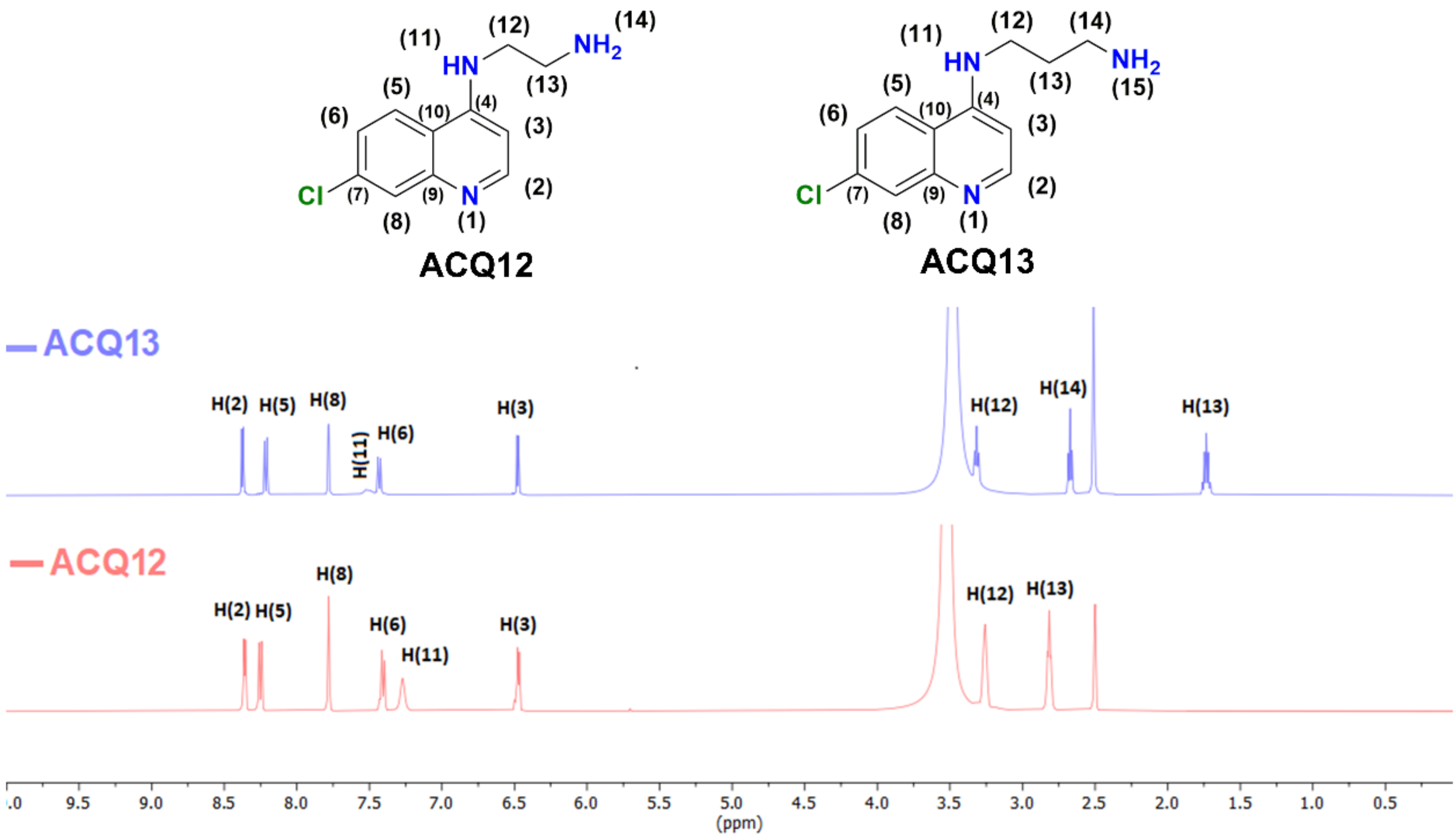

Figure S31. ^{1}H NMR (297 K, 500 MHz, DMSO-d_6) for ACQ12 and ACQ13

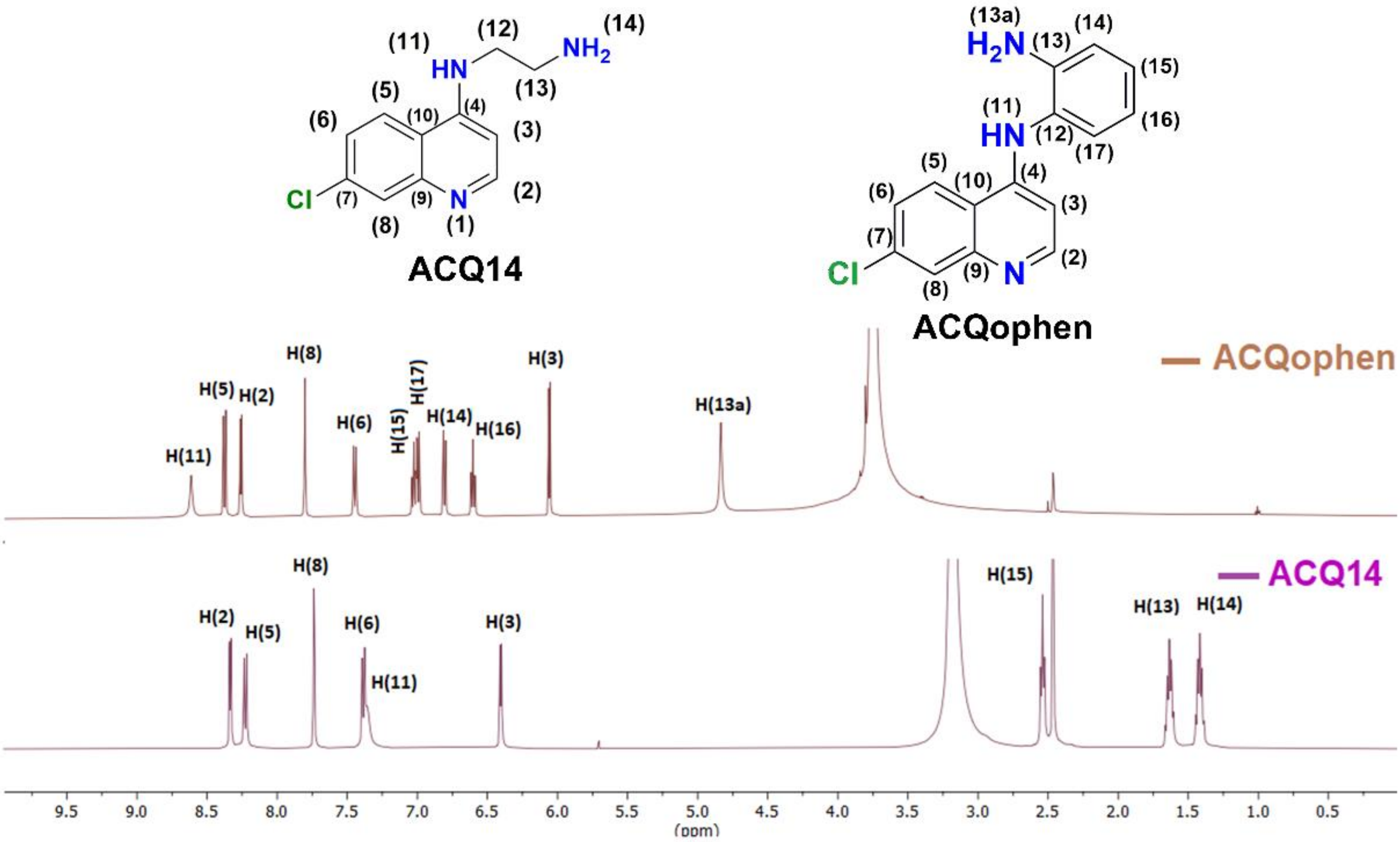

Figure S32. ^{1}H NMR (297 K, 500 MHz, DMSO-d_6) for ACQ14 and ACQophen

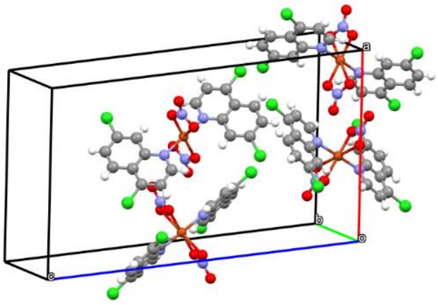

Figure S33. Unit cell for species Cu-DCQ

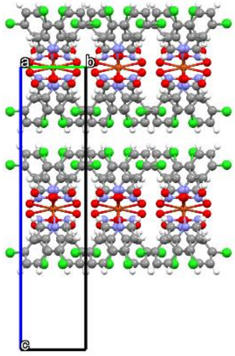

Figure S34. Supramolecular structures viewed down *a* axe for species Cu-DCQ

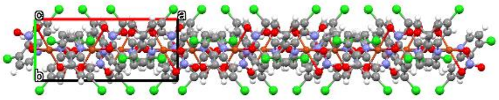

Figure S35. Supramolecular structures viewed down *b* axe for species Cu-DCQ

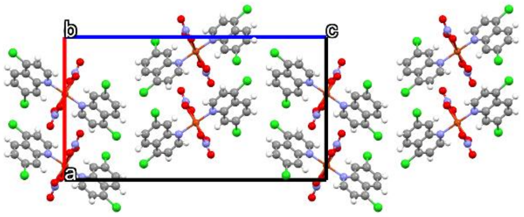

Figure S36. Supramolecular structures viewed down *c* axe for species Cu-DCQ

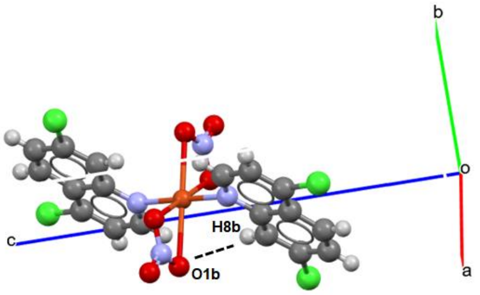

Figure S37. Intermolecular interactions for Cu-DCQ (O1b···H8b)

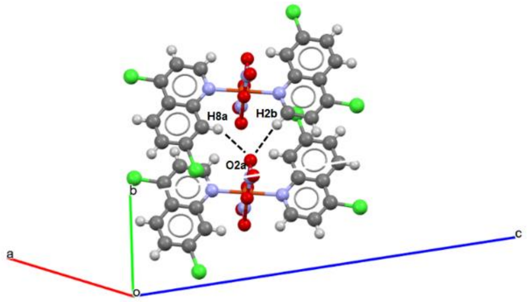

Figure S38. Intermolecular interactions for Cu-DCQ (O2a···H2b and O2a···H8a)

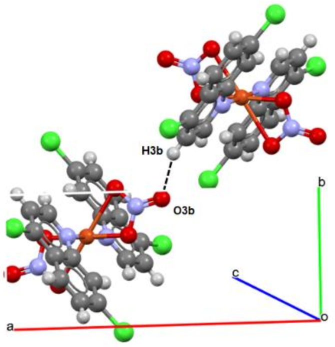

Figure S39. Intermolecular interactions for Cu-DCQ (O3b···H3b)

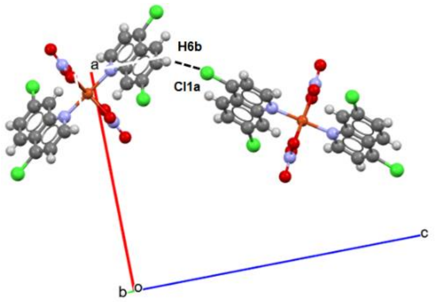

Figure S40. Intermolecular interactions for Cu-DCQ (Cl1a···H6b)

Table S1. Hydrogen bonds and interactions for Cu-DCQ

D–H···A	H···A / Å	D···A / Å	D–H···A / °
C8b–H8b···O1b	2.59(9)	3.17(4)	120.5(3)
C2b^{ii}–H2b^{ii}···O2a	2.56(4)	3.42(9)	155.3(1)
C8a^{ii}–H8a^{ii}···O2a	2.61(2)	3.37(2)	139.2(3)
C3b^{iii}–H3b^{iii}···O3b	2.38(6)	3.18(3)	143.4(5)
C6b^{i}–H6b^{i}···Cl1a	3.12(9)	3.3(40)	94.8(4)

Symmetry codes: i D–H (1-x, -y,-1/2+ z), A (x, y,z) **ii** D–H (x, -1+y,z), A (x, y, z) **iii** D–H (-1/2+x, 2–y, z), A (x, y, z)

Table S2. Fractional atomic coordinates and isotropic or equivalent isotropic displacement parameters for Cu-DCQ

Atom	x / Å×10^4	y / Å×10^4	z / Å×10^4	U_{iso}*/U_{eq} (Å^2×10^3)
Cu	-6007.4(12)	-5006(3)	-5013.8(9)	32.2(5)
N1a	-6818(7)	-5263(14)	-4365(4)	28.9(19)
N1b	-5199(7)	-4710(15)	-5663(4)	32(2)
N2a	-4549(7)	-7261(17)	-4633(4)	38(2)
N2b	-7468(7)	-2746(15)	-5403(4)	37(2)
O1a	-4787(7)	-5170(13)	-4612(4)	39.6(19)
O1b	-7237(7)	-4848(12)	-5426(4)	40.0(19)
O2a	-5131(7)	-8610(14)	-4839(4)	48(2)
O2b	-6884(7)	-1415(14)	-5200(4)	48(2)
O3a	-3759(7)	-7862(19)	-4452(5)	57(2)
O3b	-8236(7)	-2187(19)	-5592(4)	55(2)
Cl1a	-8882(3)	-6483(6)	-3021.5(14)	60.0(10)
Cl1b	-3069(3)	-3538(6)	-6980.7(13)	55.0(9)
Cl2a	-5314(3)	1627(5)	-3465.3(12)	52.0(8)
Cl2b	-6671(3)	-11614(5)	-6576.8(13)	51.2(8)
C2a	-7434(9)	-7016(19)	-4365(5)	39(2)
C2b	-4577(10)	-3010(20)	-5667(5)	42(3)
C3a	-8083(9)	-7430(20)	-3959(6)	46(3)
C3b	-3921(9)	-2550(20)	-6064(5)	43(3)
C4a	-8092(8)	-6010(20)	-3539(5)	38(2)
C4b	-3903(8)	-3950(20)	-6484(5)	39(2)
C5a	-7427(9)	-2580(20)	-3090(5)	44(3)
C5b	-4587(9)	-7361(18)	-6953(4)	39(2)
C6a	-6787(9)	-840(20)	-3087(4)	42(3)
C6b	-5227(10)	-9060(20)	-6959(4)	46(3)
C7a	-6141(9)	-620(20)	-3497(5)	40(2)
C7b	-5879(9)	-9400(20)	-6545(5)	38(2)

C8a	-6132(8)	-2030(20)	-3928(5)	37(2)
C8b	-5865(8)	-7970(20)	-6115(4)	36(2)
C9a	-6806(8)	-3823(19)	-3934(4)	33(2)
C9b	-5205(8)	-6150(20)	-6094(4)	34(2)
C10a	-7456(8)	-4142(18)	-3514(4)	34(2)
C10b	-4561(8)	-5840(20)	-6519(5)	38(2)
H2a	-7426.7	-8005.08	-4650.02	47
H2b	-4579.76	-2046.76	-5378.94	50
H3a	-8504.65	-8655.18	-3975.45	55
H3b	-3505.94	-1310.1	-6041.43	52
H5a	-7853.08	-2757.45	-2810.6	53
H5b	-4159.78	-7172.44	-7231.15	46
H6a	-6782.73	194.3	-2811.12	51
H6b	-5239.53	-10044.62	-7245.25	55
H8a	-5698.37	-1820.04	-4202.37	45
H8b	-6291.27	-8214.82	-5839.01	44

Table S3. Atomic displacement parameters for Cu-DCQ

Atom	U_{11} / Å^2×10^3	U_{22} / Å^2×10^3	U_{33} / Å^2×10^3	U_{23} / Å^2×10^3	U_{13} / Å^2×10^3	U_{12} / Å^2×10^3
Cu	36.6(8)	26.8(8)	33.1(8)	-3.4(4)	-1.9(5)	3.5(4)
N1a	39(5)	21(4)	27(4)	-1(3)	5(4)	3(3)
N1b	36(5)	27(5)	33(4)	0(3)	5(4)	-1(3)
N2a	42(5)	33(5)	40(4)	2(4)	1(4)	2(4)
N2b	39(5)	31(4)	41(5)	6(4)	1(4)	3(4)
O1a	48(5)	30(4)	41(4)	-7(3)	-14(4)	2(3)
O1b	49(5)	32(4)	39(4)	-7(3)	-8(4)	0(3)
O2a	57(5)	29(4)	58(5)	-6(4)	-1(4)	-7(3)
O2b	54(5)	32(4)	56(5)	-7(4)	-2(4)	-8(3)
O3a	46(5)	58(6)	68(6)	4(5)	-5(5)	12(4)
O3b	49(5)	57(6)	59(6)	9(5)	-12(4)	11(4)
Cl1a	65(2)	56(2)	59.3(18)	2.7(15)	20.6(16)	-13.1(15)
Cl1b	61(2)	48.1(18)	56.2(17)	5.6(13)	16.7(14)	-4.5(13)
Cl2a	65.9(19)	31.6(15)	58.4(17)	-5.9(12)	-6.8(14)	-10.5(12)
Cl2b	64.9(19)	32.5(16)	56.1(17)	-6.7(13)	-4.6(14)	-10.6(12)
C2a	44(6)	25(5)	49(6)	0(4)	-5(5)	-3(4)
C2b	57(7)	30(6)	39(5)	-3(4)	-1(5)	0(5)
C3a	47(7)	32(6)	57(7)	-5(5)	1(5)	-4(5)
C3b	50(7)	25(6)	55(7)	6(5)	-9(5)	-3(5)
C4a	39(6)	29(5)	46(6)	12(5)	3(5)	0(4)
C4b	45(7)	31(6)	41(6)	16(5)	1(4)	0(4)
C5a	52(7)	41(6)	41(5)	-4(5)	0(5)	9(5)
C5b	54(7)	29(5)	33(5)	-4(4)	5(4)	3(4)
C6a	62(7)	25(6)	40(5)	-2(5)	-4(5)	3(5)
C6b	66(8)	37(6)	34(5)	-10(5)	-6(5)	6(5)
C7a	49(6)	23(5)	48(6)	-5(5)	-12(5)	-1(5)
C7b	47(6)	22(5)	46(6)	2(5)	-2(5)	3(5)
C8a	37(6)	31(6)	44(6)	2(5)	2(4)	5(4)
C8b	38(5)	37(6)	34(5)	2(4)	-5(4)	-4(4)
C9a	38(5)	24(5)	37(5)	-3(4)	-2(4)	8(4)
C9b	40(5)	31(6)	31(5)	-3(4)	-4(4)	3(4)
C10a	42(5)	22(5)	37(5)	2(4)	-1(4)	8(4)
C10b	41(6)	28(6)	44(6)	5(5)	-10(5)	7(5)

Table S4. Bond Lenghts for Cu-DCQ

Atoms		Length /Å	Atoms		Length /Å
Cu	O1A	1.978(9)	C2A	C3A	1.393(19)

Cu	O1B	2.004(9)	C10B	C9B	1.414(17)
Cu	N1A	2.004(9)	C10B	C4B	1.441(18)
Cu	N1B	2.006(9)	C10B	C5B	1.424(16)
Cl2B	C7B	1.707(13)	C8A	C9A	1.409(17)
Cl2A	C7A	1.750(14)	C8A	C7A	1.379(19)
Cl1B	C4B	1.730(12)	C10A	C5A	1.418(16)
Cl1A	C4A	1.735(12)	C10A	C9A	1.411(16)
O1A	N2A	1.277(13)	C10A	C4A	1.412(18)
O1B	N2B	1.281(12)	C5A	C6A	1.357(19)
O3A	N2A	1.239(14)	C2B	C3B	1.387(19)
O2A	N2A	1.248(14)	C3A	C4A	1.358(19)
O2B	N2B	1.241(13)	C9B	C8B	1.410(16)
N1A	C2A	1.339(15)	C7B	C6B	1.405(18)
N1A	C9A	1.388(14)	C7B	C8B	1.379(18)
O3B	N2B	1.212(13)	C6A	C7A	1.382(19)
N1B	C2B	1.319(17)	C4B	C3B	1.354(18)
N1B	C9B	1.388(14)	C6B	C5B	1.338(19)

Table S5. Bond Angles for Cu-DCQ

Atom			Angle / °	Atom			Angle / °
O1A	Cu	O1B	179.5(5)	C4A	C10A	C5A	123.8(11)
O1A	Cu	N1A	92.8(4)	C6A	C5A	C10A	120.8(12)
O1A	Cu	N1B	87.3(4)	N1B	C2B	C3B	125.7(12)
O1B	Cu	N1B	92.2(4)	C4A	C3A	C2A	119.0(11)
N1A	Cu	O1B	87.7(4)	N1B	C9B	C10B	121.3(11)
N1A	Cu	N1B	179.3(4)	N1B	C9B	C8B	120.0(10)
N2A	O1A	Cu	104.3(7)	C8B	C9B	C10B	118.6(10)
N2B	O1B	Cu	103.5(6)	C6B	C7B	Cl2B	119.1(10)
O3A	N2A	O1A	119.2(10)	C8B	C7B	Cl2B	120.8(9)
O3A	N2A	O2A	123.1(10)	C8B	C7B	C6B	120.1(12)
O2A	N2A	O1A	117.7(10)	C5A	C6A	C7A	119.4(12)
C2A	N1A	Cu	114.5(8)	C10B	C4B	Cl1B	119.2(9)
C2A	N1A	C9A	118.6(10)	C3B	C4B	Cl1B	120.3(9)
C9A	N1A	Cu	126.8(8)	C3B	C4B	C10B	120.5(11)
C2B	N1B	Cu	115.9(8)	N1A	C9A	C8A	118.3(10)
C2B	N1B	C9B	117.5(10)	N1A	C9A	C10A	120.7(10)
C9B	N1B	Cu	126.5(8)	C8A	C9A	C10A	121.0(10)
O2B	N2B	O1B	117.8(9)	C8A	C7A	Cl2A	119.2(11)
O3B	N2B	O1B	117.7(10)	C8A	C7A	C6A	123.3(12)
O3B	N2B	O2B	124.5(10)	C6A	C7A	Cl2A	117.4(10)
N1A	C2A	C3A	123.1(11)	C10A	C4A	Cl1A	119.0(9)
C9B	C10B	C4B	117.0(10)	C3A	C4A	Cl1A	120.4(10)
C9B	C10B	C5B	119.7(11)	C3A	C4A	C10A	120.6(11)
C5B	C10B	C4B	123.3(11)	C4B	C3B	C2B	117.9(11)
C7A	C8A	C9A	117.1(11)	C5B	C6B	C7B	121.5(11)
C9A	C10A	C5A	118.3(11)	C7B	C8B	C9B	120.2(11)
C9A	C10A	C4A	117.9(10)	C6B	C5B	C10B	119.8(11)

Table S6. Torsion Angles for Cu-DCQ

Atoms				Angle / °	Atoms				Angle / °
Cu	O1A	N2A	O3A	73.8(9)	C5A	C6A	C7A	C8A	2.6(19)
Cu	O1A	N2A	O2A	6.0(12)	C2B	N1B	C9B	C10B	0.8(16)
Cu	O1B	N2B	O2B	6.7(12)	C2B	N1B	C9B	C8B	-178.9(11)
Cu	O1B	N2B	O3B	-174.7(9)	C9B	N1B	C2B	C3B	-0.4(19)

Cu	N1A	C2A	C3A	177.5(10)	C9B	C10B	C4B	Cl1B	177.7(8)
Cu	N1A	C9A	C8A	-3.2(15)	C9B	C10B	C4B	C3B	-0.8(16)
Cu	N1A	C9A	C10A	176.7(7)	C9B	C10B	C5B	C6B	0.8(17)
Cu	N1B	C2B	C3B	177.1(10)	C7B	C6B	C5B	C10B	-0.2(19)
Cu	N1B	C9B	C10B	-176.5(8)	C4B	C10B	C9B	N1B	-0.2(16)
Cu	N1B	C9B	C8B	3.8(15)	C4B	C10B	C9B	C8B	179.5(10)
Cl2B	C7B	C6B	C5B	-179.6(9)	C4B	C10B	C5B	C6B	-179.2(11)
Cl2B	C7B	C8B	C9B	179.8(9)	C9A	N1A	C2A	C3A	1.9(17)
Cl1B	C4B	C3B	C2B	-177.4(9)	C9A	C8A	C7A	Cl2A	179.6(8)
N1A	C2A	C3A	C4A	-0.8(19)	C9A	C8A	C7A	C6A	-1.6(18)
N1B	C2B	C3B	C4B	-0.5(19)	C9A	C10A	C5A	C6A	-0.5(17)
N1B	C9B	C8B	C7B	179.3(10)	C9A	C10A	C4A	Cl1A	178.7(8)
C2A	N1A	C9A	C8A	177.6(10)	C9A	C10A	C4A	C3A	-1.0(17)
C2A	N1A	C9A	C10A	-2.6(16)	C7A	C8A	C9A	N1A	179.4(10)
C2A	C3A	C4A	Cl1A	-179.4(10)	C7A	C8A	C9A	C10A	-0.4(17)
C2A	C3A	C4A	C10A	0.3(19)	C4A	C10A	C5A	C6A	178.9(12)
C10B	C9B	C8B	C7B	-0.4(17)	C4A	C10A	C9A	N1A	2.2(15)
C10B	C4B	C3B	C2B	1.1(17)	C4A	C10A	C9A	C8A	-178.0(10)
C10A	C5A	C6A	C7A	-1.4(18)	C6B	C7B	C8B	C9B	0.9(17)
C5A	C10A	C9A	N1A	-178.4(10)	C8B	C7B	C6B	C5B	-0.7(19)
C5A	C10A	C9A	C8A	1.4(16)	C5B	C10B	C9B	N1B	179.8(10)
C5A	C10A	C4A	Cl1A	-0.7(16)	C5B	C10B	C9B	C8B	-0.5(16)
C5A	C10A	C4A	C3A	179.6(11)	C5B	C10B	C4B	Cl1B	-2.3(16)
C5A	C6A	C7A	Cl2A	-178.6(9)	C5B	C10B	C4B	C3B	179.2(10)

Document origin: *publCIF* [Westrip, S.P. *J. Apply. Cryst.* 43 (2010) 920–925].

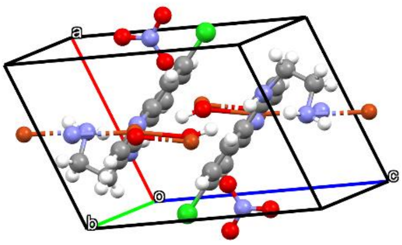

Figure S41. Unit cell for species Cu-ACQ12

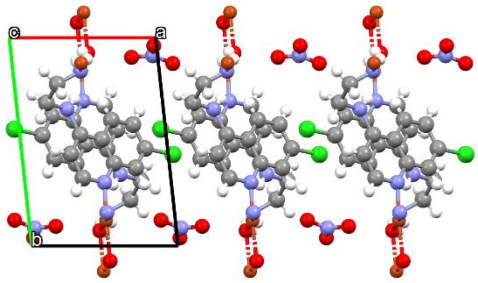

Figure S42. Supramolecular structures viewed down *a* axe for species Cu-ACQ12

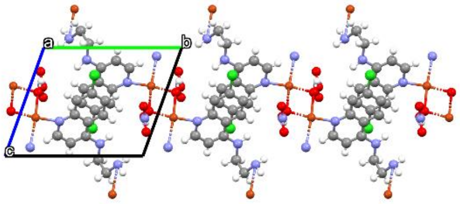

Figure S43. Supramolecular structures viewed down *b* axe for species Cu-ACQ12

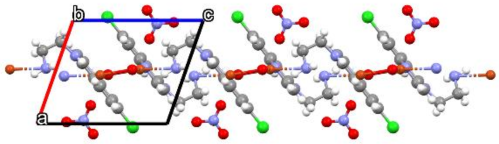

Figure S44. Supramolecular structures viewed down *c* axe for species Cu-ACQ12

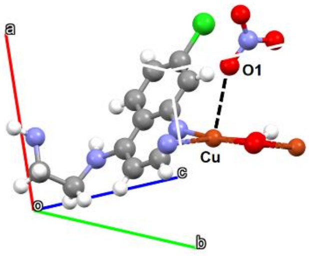

Figure S45. Intermolecular interactions for Cu-ACQ12 (Cu･･･O1)

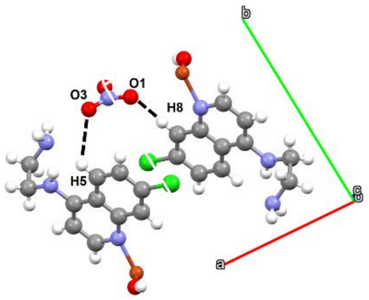

Figure S46. Intermolecular interactions for Cu-ACQ12 (O1···H8 and O3···H5)

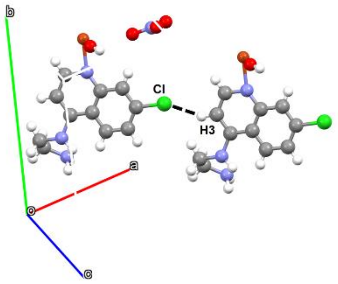

Figure S47. Intermolecular interactions for Cu-ACQ12 (Cl···H3)

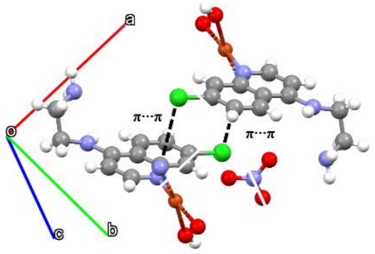

Figure S48. Intermolecular interactions for Cu-ACQ12 (π···π)

Table S7. Hydrogen bonds and interactions for Cu-ACQ12

D–H···A	H···A / Å	D···A / Å	D–H···A / °
Cu···O1	–	2.89(8)	–
C8–H8···O1	2.30(1)	3.15(2)	148.(70)
$C5^{i}$–$H5^{i}$···O3	2.8(80)	3.47(6)	121.7(9)
$C3^{ii}$–$H3^{ii}$···Cl	2.82(4)	3.69(3)	152.7(2)
$\pi^{iii}\cdots\pi$	–	3.18(9)	–

Symmetry codes: i D–H (2-x, 1-y, 1-z), A (x, y, z) **ii** D–H (1+x, y, 1+z), A (x, y, z) **iii** D–H (2-x, 1-y, 1-z), A (x, y, z)

Table S8. Atomic Positional Parameters for Cu-ACQ12

Atom	*x* / Å	*y* / Å	*z* / Å
Cu	0.47(4)	0.12(02)	0.640(6)
N	1.03260	0.91353	0.65052
N1	0.53817	0.69590	0.31710
N11	0.34268	0.34342	0.11034
N14	0.45826	0.16534	-0.08537
O1	0.91010	0.86523	0.53782
O2	1.00868	0.96425	0.77292
O3	1.17902	0.91110	0.64083

Cl	1.02689	0.56331	0.74026
C2	0.39442	0.67320	0.18693
C3	0.33066	0.55824	0.11950
C4	0.41066	0.46599	0.18224
C5	0.63440	0.39644	0.37515
C6	0.77816	0.41914	0.50532
C7	0.84191	0.53410	0.57276
C8	0.76192	0.62635	0.51002
C9	0.61816	0.60365	0.37984
C10	0.55441	0.48869	0.31241
C12	0.20241	0.31455	-0.03657
C13	0.28340	0.22339	-0.15705
H2	0.33854	0.73764	0.14310
H3	0.23025	0.54238	0.02857
H5	0.80645	0.70666	0.55712
H6	0.83404	0.35470	0.54915
H8	0.58987	0.31613	0.32805
H11	0.40361	0.29350	0.17375
H12a	0.15049	0.39023	-0.05916
H12b	0.11169	0.27688	-0.03507
H13a	0.29727	0.26811	-0.21197
H13b	0.20564	0.15965	-0.22451
H14a	0.52004	0.23053	-0.01367
H14b	0.43916	0.10622	-0.06116

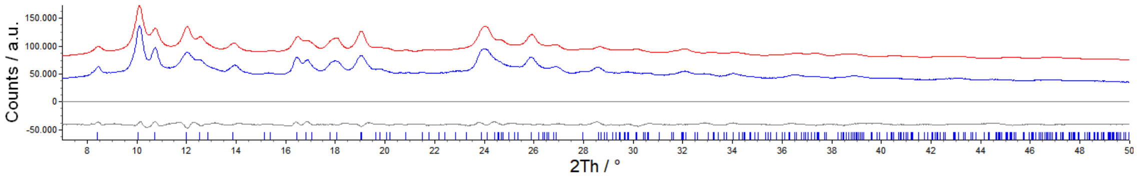

Figure S49. Final Rietveld refinement plot for Cu-ACQ12, experimental, blue, calculated, red, with the difference plot, gray, and peak markers at the bottom.

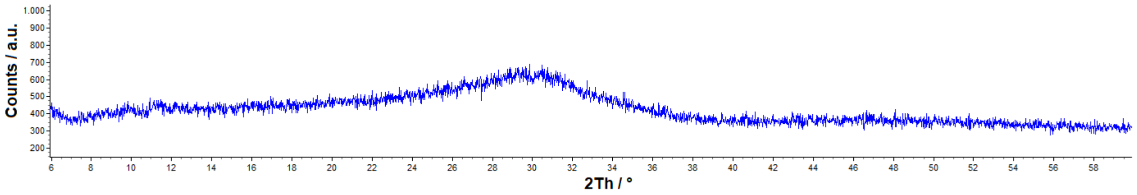

Figure S50. Experimental X ray powder diffraction pattern for Cu-ACQ13.

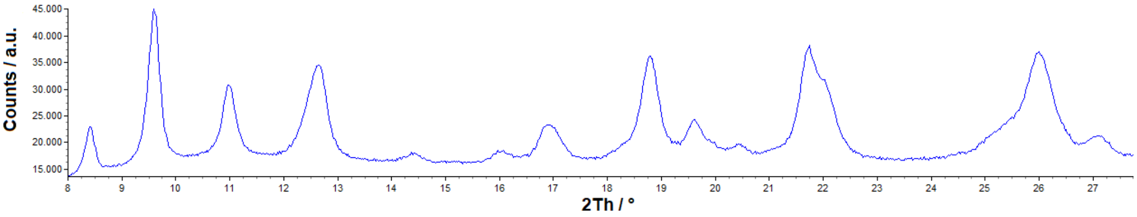

Figure S51. Experimental X ray powder diffraction pattern for Cu-ACQ14.

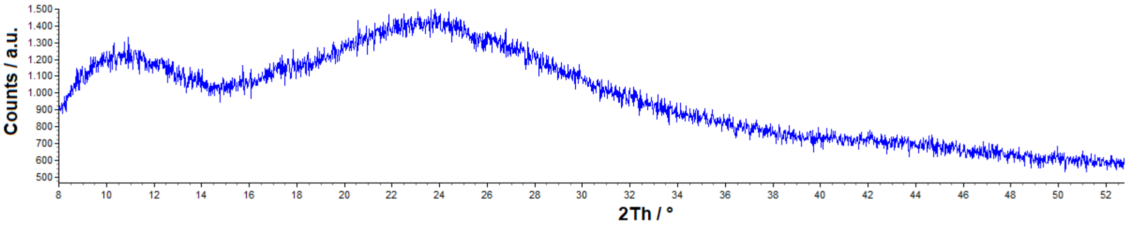

Figure S52. Experimental X ray powder diffraction pattern for complex Cu-ACQophen.

Table S9. Optimized geometries of the Cu-DCQ, Cu-ACQ12, and Cu-ACQ13 complexes obtained at the DFT level, showing the main Cu–N and Cu–O coordination bonds lengths (in Å)

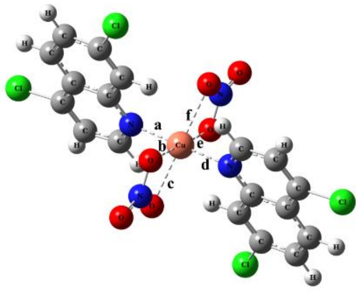

Cu-DCQ

Legend	Bond length (Å)
**(a)** Cu35-N15	2.01
**(b)** Cu35-O39	2.05
**(c)** Cu35-O38	2.31
**(d)** Cu35-N32	2.01
**(e)** Cu35-O36	2.05
**(f)** Cu35-O37	2.31

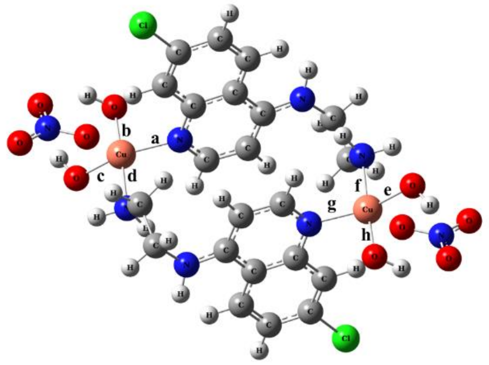

Cu-ACQ12

Legend	Bond length (Å)
**(a)** Cu7-N47	1.97
**(b)** Cu7-O8	1.78
**(c)** Cu7-O1	1.79
**(d)** Cu7-N21	1.97
**(e)** Cu43-O9	1.79
**(f)** Cu43-N52	1.97
**(g)** Cu43-N16	1.97
**(h)** Cu43-O44	1.78

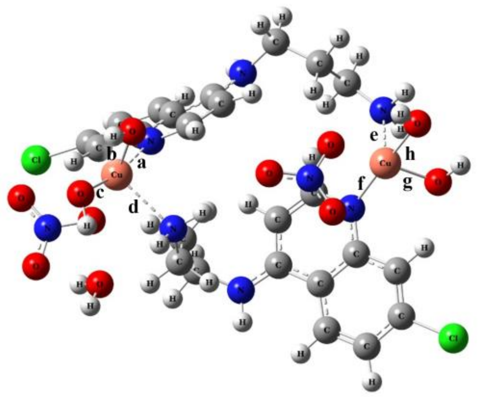

Cu-ACQ13

Legend	Bond length (Å)
**(a)** Cu40-N16	1.99
**(b)** Cu40-O41	1.79
**(c)** Cu40-O9	1.79
**(d)** Cu40-N49	2.35
**(e)** Cu7-N21	2.38
**(f)** Cu7-N44	1.94
**(g)** Cu7-O8	1.79
**(h)** Cu7-O1	1.80

Table S9. Optimized geometries of the Cu-ACQ14 and Cu-ACQophen complexes obtained at the DFT level, showing the main Cu–N and Cu–O coordination bonds lengths (in Å)

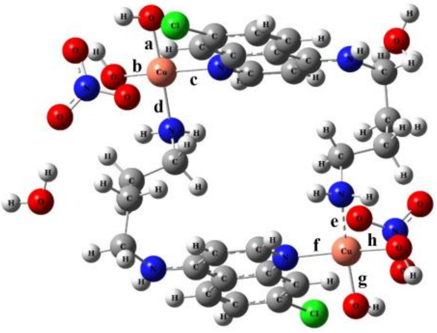

Cu-ACQ14

Legend	Bond length (Å)
**(a)** Cu7-O8	1.78
**(b)** Cu7-O1	1.79
**(c)** Cu7-N21	1.98
**(d)** Cu7-N44	1.96
**(e)** Cu40-N49	2.00
**(f)** Cu40-N16	1.97
**(g)** Cu40-O41	1.79
**(h)** Cu40-O9	1.80

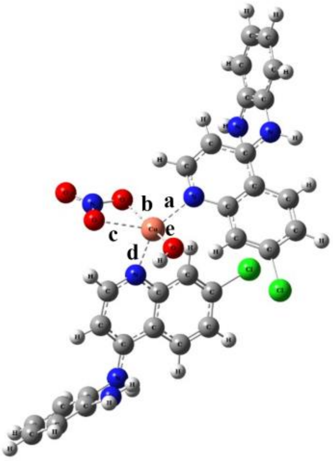

Cu-ACQOphen

Legend	Bond length (Å)
**(a)** Cu67-N15	2.10
**(b)** Cu67-O66	2.04
**(c)** Cu67-O65	2.39
**(d)** Cu67-N46	2.10
**(e)** Cu67-O68	1.85

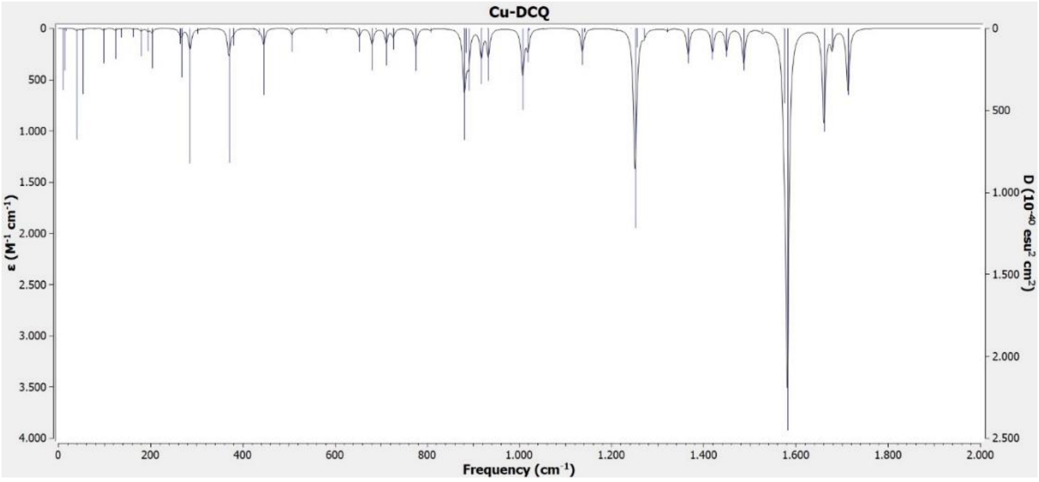

Figure S53. Infrared spectra for complex Cu-DCQ theoretically calculated at level M062X/6-31G.

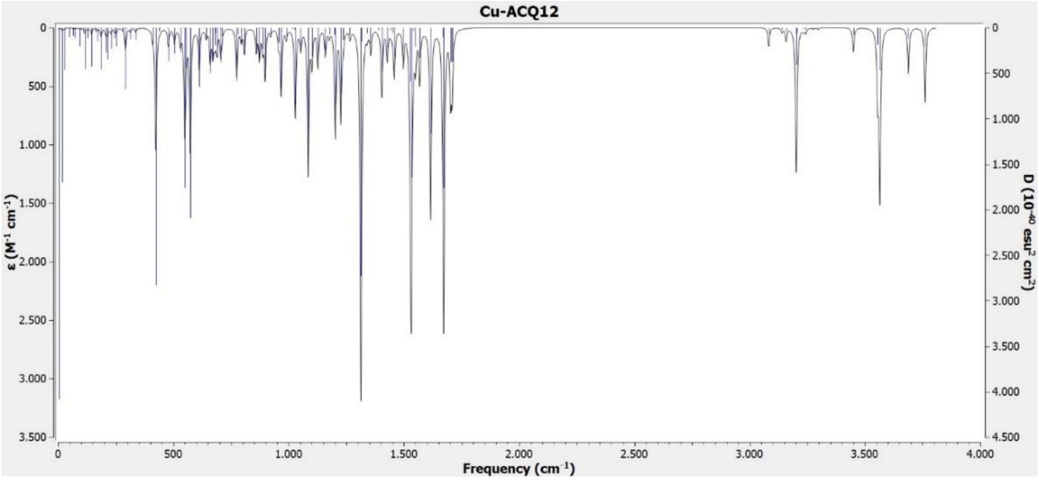

Figure S54. Infrared spectra for complex Cu-ACQ12 theoretically calculated at level M062X/6-31G

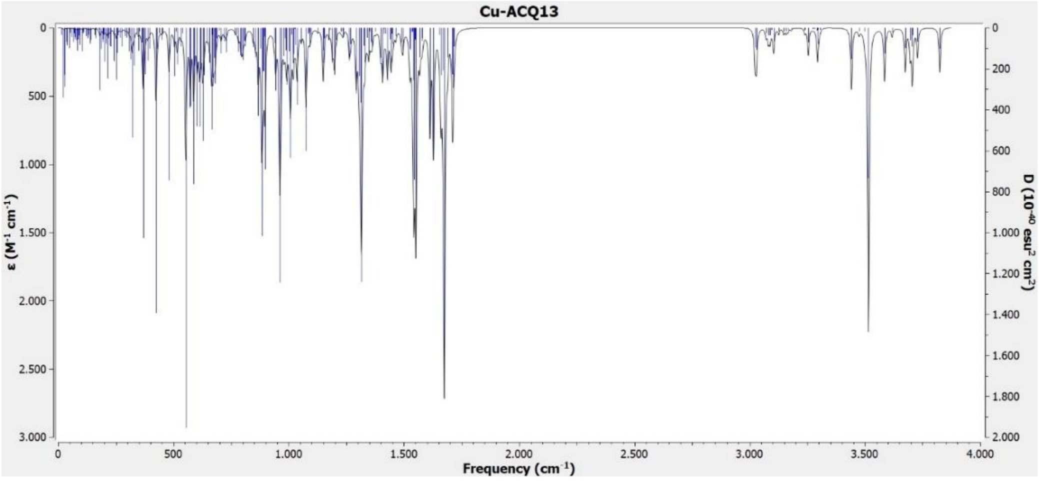

Figure S55. Infrared spectra for complex Cu-ACQ13 theoretically calculated at level M062X/6-31G

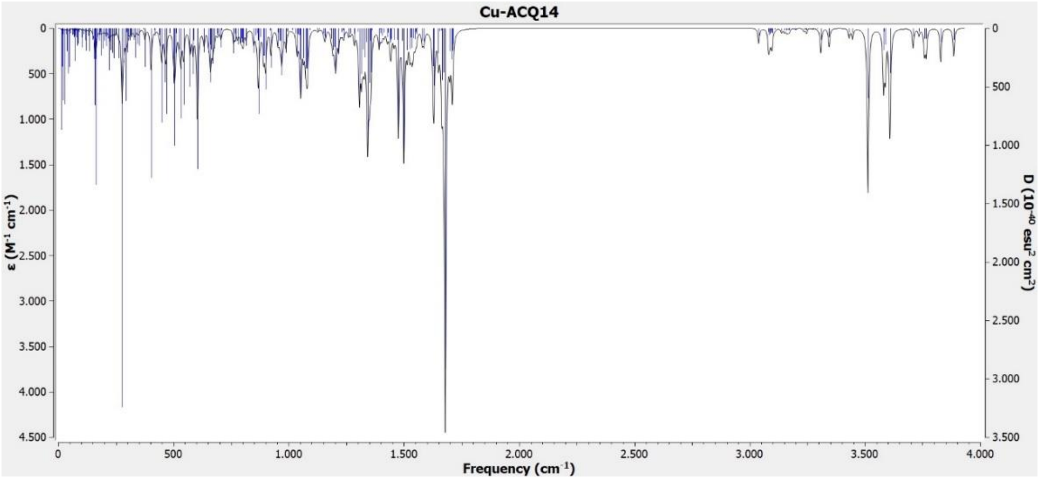

Figure S56. Infrared spectra for complex Cu-ACQ14 theoretically calculated at level M062X/6-31G

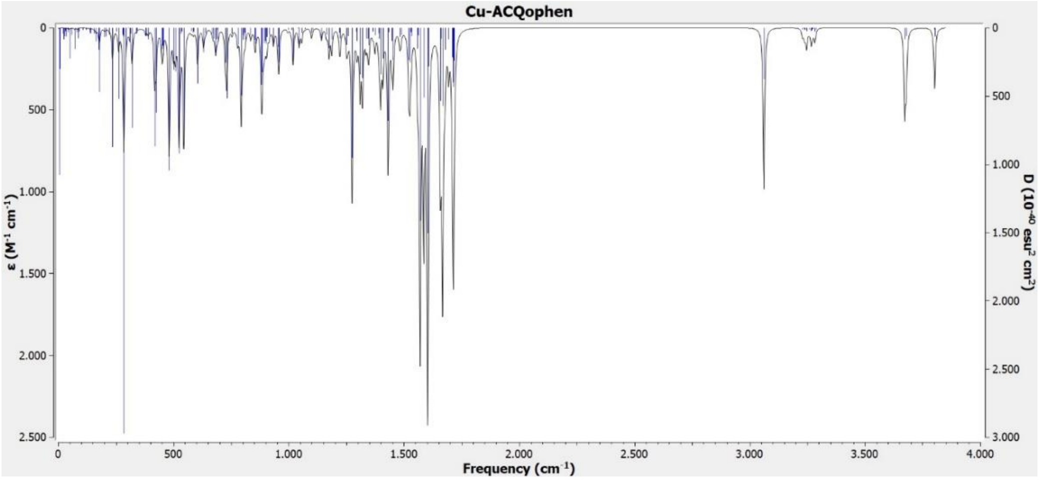

Figure S57. Infrared spectra for complex Cu-ACQophen theoretically calculated at level M062X/6-31G

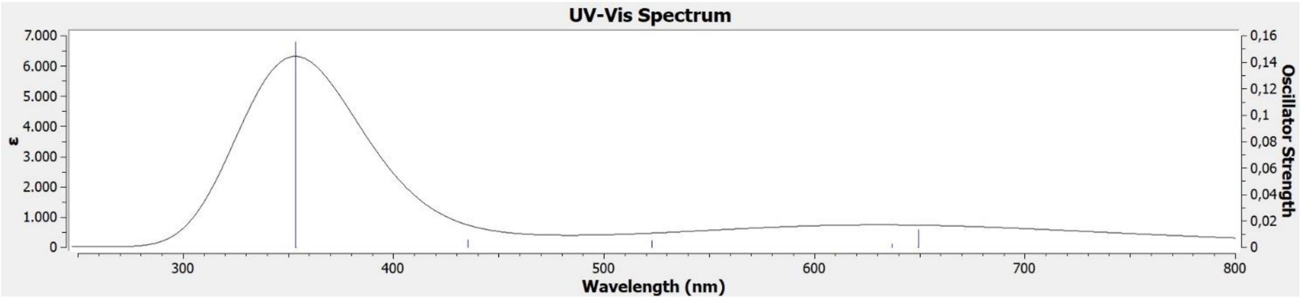

Figure S58. UV-Vis spectra for complex Cu-ACQ12 theoretically calculated at level M062X/6-311G(d,p)/6-31G/SMD(DMSO).

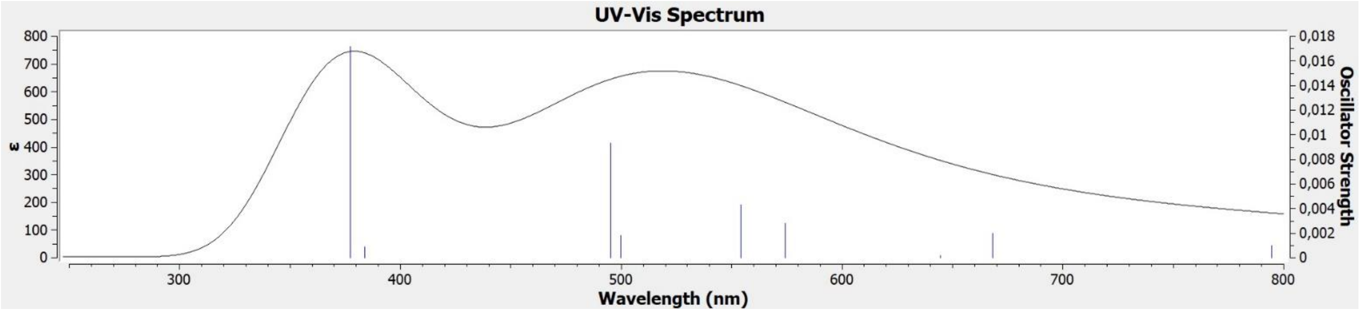

Figure S59. UV-Vis spectra for complex Cu-ACQ13 theoretically calculated at level M062X/6-311G(d,p)/6-31G/SMD(DMSO).

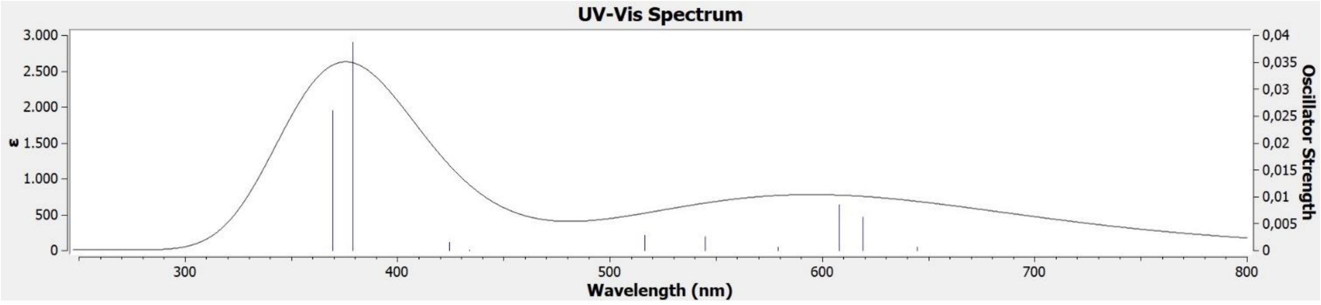

Figure S60. UV-Vis spectra for complex Cu-ACQ14 theoretically calculated at level M062X/6-311G(d,p)/6-31G/SMD(DMSO).
